# Reference-Guided De Novo Genome Assembly of the Flour Beetle *Tribolium freemani*

**DOI:** 10.3390/ijms23115869

**Published:** 2022-05-24

**Authors:** Marin Volarić, Evelin Despot-Slade, Damira Veseljak, Nevenka Meštrović, Brankica Mravinac

**Affiliations:** Division of Molecular Biology, Ruđer Bošković Institute, Bijenička Cesta 54, 10000 Zagreb, Croatia; marin.volaric@irb.hr (M.V.); evelin.despot.slade@irb.hr (E.D.-S.); damira.veseljak@irb.hr (D.V.); nevenka@irb.hr (N.M.)

**Keywords:** *Tribolium freemani*, de novo genome assembly, mitogenome, repetitive DNA, sibling species, the flour beetle, PacBio HiFi

## Abstract

The flour beetle *Tribolium freemani* is a sibling species of the model organism and important pest *Tribolium castaneum*. The two species are so closely related that they can produce hybrid progeny, but the genetic basis of their differences has not been revealed. In this work, we sequenced the *T. freemani* genome by applying PacBio HiFi technology. Using the well-assembled *T. castaneum* genome as a reference, we assembled 262 Mb of the *T. freemani* genomic sequence and anchored it in 10 linkage groups corresponding to nine autosomes and sex chromosome X. The assembly showed 99.8% completeness of conserved insect genes, indicating a high-quality reference genome. Comparison with the *T. castaneum* assembly revealed that the main differences in genomic sequence between the two sibling species come from repetitive DNA, including interspersed and tandem repeats. In this work, we also provided the complete assembled mitochondrial genome of *T. freemani*. Although the genome assembly needs to be ameliorated in tandemly repeated regions, the first version of the *T. freemani* reference genome and the complete mitogenome presented here represent useful resources for comparative evolutionary studies of related species and for further basic and applied research on different biological aspects of economically important pests.

## 1. Introduction

The decades-long eagerness of scientists to obtain reference genomes for species of interest has been inspired by the reliance that the availability of whole-genome data of various species will further our biological understanding and provide the infrastructure for addressing comprehensively questions on evolution, ecology, conservation, biomedicine, agriculture, and many other areas vital to sustainable life on Earth. The rapid development of third-generation sequencing technologies and tailored bioinformatics tools that have experienced an explosive boom in the last decade has enabled the deciphering of genomes of different species in a relatively short time and at affordable prices [1]. The two cutting-edge technologies that currently dominate the field of genome sequencing have been introduced by PacBio (Pacific Biosciences, Menlo Park, CA, USA) and ONT (Oxford Nanopore Technologies, Oxford, UK). In terms of sequence accuracy and read length, both approaches have their advantages, but at the moment, PacBio HiFi sequencing represents an optimal compromise ensuring >99.9% accuracy for reads up to 25 kb [2]. Consequently, PacBio HiFi data are included in the current best-practice genome assembly guidelines of different genome sequencing consortia, from extensive ones such as Earth BioGenome Project [3] to more specialized ones, such as the Ag100Pest Initiative, which aims to generate reference genome assemblies for agricultural pest arthropods [4].

One million insect species make the class Insecta the most numerous group of the kingdom Animalia, and beetles with >387,000 species represent not only the largest insect order Coleoptera but also account for 25% of all described animal life forms [5]. Beetles have also been acknowledged as excellent model organisms in life sciences research through different biological, environmental, and biomedical studies [6]. Despite the impressive abundance of described beetle species with a potentially much higher number of undiscovered ones, a surprisingly small number of beetle genomes have been sequenced so far, and in terms of available genome assemblies relative to species richness, Coleoptera is heavily underrepresented [7]. The importance of high-quality genome reference sequences has been recognized by the insect research community, which strongly advocates sequencing a large number of coleopteran genomes and making these data publicly accessible [4,8].

**Figure 1:**
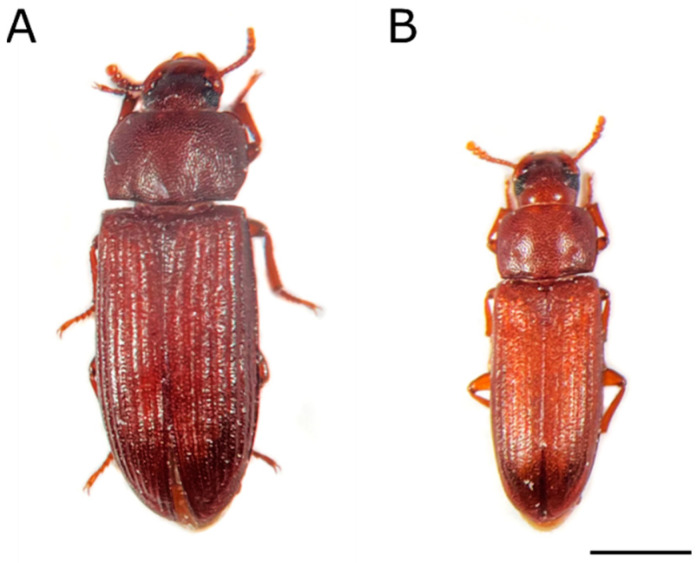
Dorsal view of the Kashmir flour beetle *Tribolium freemani* (**A**) and the red flour beetle *Tribolium castaneum* (**B**). Scale bar = 1 mm.

The representative species of Coleoptera is the red flour beetle, *Tribolium castaneum*, an economically important pest of stored agricultural products. In addition to its cosmopolitan distribution, *T. castaneum* has also been present in laboratory research for half a century [9]. As a coleopteran model organism, it is the first beetle to have its genome sequenced [10], and the genome assembly has recently been upgraded, providing a new official gene set for *T. castaneum* [11]. The Kashmir flour beetle, *Tribolium freemani,* is a sibling species of *T. castaneum* (Figure 1). The first recorded specimen of *T. freemani*, an adult female, was collected at Hispar in Kashmir (India) around 1893 and deposited in the British Museum (nowadays The Natural History Museum), but the specimen was described as a new species five decades later by British entomologist H. E. Hinton [12]. It took the next 30 years to rediscover the living adults of *T. freemani*, which were found in Japan in a shipment of corn imported from Brazil [13]. The live adults captured from that shipment were propagated into a stable culture [13], which became the start-up stock for future laboratory research worldwide.

Among 36 species of the genus *Tribolium*, Hinton declared *T. freemani* the most related to *T. castaneum* based on morphological characteristics [12]. Notwithstanding the considerable difference in body size (Figure 1), the experiments of crossing *T. freemani* with *T. castaneum* showed that these two species are capable of copulating and producing progeny [13]. Although the hybrid offspring are sterile [13,14,15], the fact that the two species can hybridize speaks in favor of their genetic similarity. Based on the relative DNA content in Feulgen-stained spermatids, it was estimated that both *T. castaneum* and *T. freemani* have genomes of relatively similar size, 200 Mb and 230 Mb, respectively [16], with comparable karyotypes composed of 20 chromosomes and a 9 + Xy_p_ meioformula [17]. Intriguingly, the genomes of the two species are overrun with two unrelated species-specific satellite DNAs, tandemly repeated sequences that mainly build heterochromatic blocks of centromeric and pericentromeric chromosomal regions. According to blot hybridization experiments, 17% of the *T. castaneum* genome is made up of satellite DNA TCAST [18], while 31% of the *T. freemani* genome is comprised of satellite DNA TFREE [19]. TCAST and TFREE satellite DNAs share no similarity in the nucleotide sequence, also showing a notable difference in the repeat unit length (360 bp vs. 166 bp, respectively).

The goal of this work was to sequence the *T. freemani* genome by PacBio HiFi technology to obtain highly accurate long reads and to generate the *T. freemani* genome assembly by using the *T. castaneum* genome as a reference. By comparing the genome assemblies of the two sibling species, we aspired to identify the major genomic differences between these highly repetitive and thus assembling extraordinary challenging genomes. In addition to the nuclear DNA assembly, we also aimed to supplement the *T. freemani* reference genome with the complete mitochondrial DNA sequence.

## 2. Results

### 2.1. Genome Size Evaluation

By sequencing *T. freemani* genomic DNA, we yielded 23.8 Gb of the total sequence contained in 1,617,087 HiFi reads. First, we applied a computational approach to estimate genome size and genome repetitiveness based on k-mer frequencies in the input set of all HiFi reads. There are several programs developed for this purpose, and we tested k-mer frequencies calculated by Jellyfish with GenomeScope [20], findGSE [21], and CovEST [22]. In addition to testing different algorithms, we also tested different k-mer sizes since very repetitive genomes could benefit from larger k-mers [20]. As shown in Table 1, genome size prediction varied considerably with the program used and the k-mer sizes. FindGSE predicted a larger genome size than GenomeScope, with CovEST estimates being even larger. It has to be stressed that CovEST offers different models. We run CovEST with a repeat model (CovEST RE), which assumes that certain k-mer sequences will have drastically increased occurrences, so the model tries to include them in the final prediction rather than ignore or collapse them. Due to the assumed high repetitiveness of the *T. freemani* genome, we tend to favor measurements obtained by CovEST RE analysis, thus estimating that the haploid genome size for *T. freemani* could correspond approximately to 320 Mb, an average value of CovEST RE estimates (Table 1). In addition, the repeat ratio in HiFi data was estimated in the range of 29–33% according to GenomeScope and findGSE programs (Table 1), confirming that a high presence of repetitive sequences should be expected in the *T. freemani* genome.

### 2.2. Genome Assembly

According to the estimated *T. freemani* genome size of 320 Mb, 23.8 Gb of raw sequencing data (Table 2, left) correspond to approximate 74.4× genome coverage. A total of 23.8 Gb was used for genome assembly construction with the hifiasm assembler [23]. The initial hifiasm output resulted in 679 contigs and 465.8 Mb with an N50 of 5.5 Mb (Table 2, middle). Since hifiasm output was approximately 150 Mb longer than the estimated genome size, we suspected that the difference could be due to the software’s inability to properly assemble highly repetitive regions. For this reason, we inspected 679 contigs of hifiasm output and found that 67% of the contigs (455 out of 679) contain tandem repeats of the major satellite DNA TFREE. Moreover, TFREE makes up over 50% of the total sequence length in 345 contigs (Table S1).

Due to the high repetitive DNA content in the majority of contigs, in order to minimize the inaccuracies that tandem repeats introduce to genome assembling, we decided to filter out and keep for further assembly only the contigs that contained substantial genetic information defined as a set of uniquely mappable features, UMFs (see Materials and Methods Section 4.7). For this purpose, we took advantage of the fact that the closely related species *T. castaneum* has the well-curated assembly Tcas5.2 and annotated official gene set OGS3 [11]. First, we used Liftoff to select the hifiasm output contigs that contained UMFs from the OGS3. We found that only 141 contigs contained UMFs from Tcas5.2 (Table S1). Out of 141 contigs, 110 had more than 10 UMFs (Table S1), a benchmark that we deemed sufficient for accurate mapping in subsequent steps. Indicatively, 99.95% of the total detected UMFs are present in 110 contigs selected for further assembling, while only 0.05% of UMFs are contained in 569 discarded contigs (Table S1). As shown in Figure S1, 569 discarded contigs are predominately made up of the TFREE satellite DNA. Next, by using 23.8 Gb of *T. freemani* HiFi reads, we filled in the holes of the discontinuous assembly Tcas5.2 to prevent fragmenting using the TGS-GapCloser tool with minimap2 parameters set to allow high sequence divergence. After the gap-filling step, we used the RagTag tool [24] to orient 110 previously selected, UMF-enriched contigs into 10 linkage groups corresponding to the Tcas5.2 chromosome-level linkage groups. Out of 110 contigs, 99 contigs (261.8 Mb) successfully orientated into 10 *f*LGs (abbreviation for *T. freemani* linkage groups), and 11 contigs (7.2 Mb) remained unplaced (Table S2) based on mapping and/or orientation confidence scores (Table S3).

**Figure 2:**
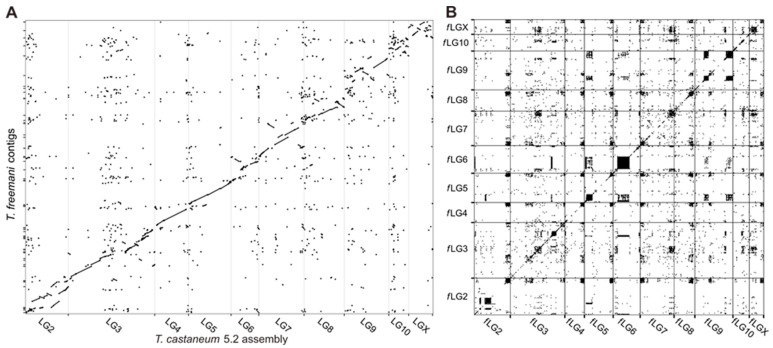
Dot plot visualization of genome-to-genome alignment produced with dotPlotly. (**A**) Comparison of *T. freemani* contigs with 10 linkage groups (LGs) representing chromosomes in the *T. castaneum* reference assembly Tcas5.2. (**B**) A self-to-self alignment of *T. freemani* assembly Tfree1.0. Abbreviations *f*LG stand for *T. freemani* linkages groups obtained by the reference-guided orientation of contigs.

The structural contiguity of the obtained *T. freemani* assembly, named Tfree1.0, was analyzed by dot plot (Figure 2). A comparison of Tfree1.0 with Tcas5.2 assembly showed that the majority of *T. freemani* contigs align with reference (Figure 2A). Furthermore, the alignment of Tfree1.0 assembly on itself (Figure 2B) reveals a higher level of self-similarity with medium to large dark blocks representing regions of repetitive sequences retained in 10 *f*LGs.

To validate the information present in the primary hifiasm output (679 contigs) but lost in the Tfree1.0 assembly (110 contigs), we plotted all hifiasm contigs against the final assembly (Figure S2). The dot plot analysis showed that the contigs present in the hifiasm output have been successfully mapped to the Tfree1.0 assembly, proving that very little to no non-repetitive information was lost.

A comparison of the size difference of the assembled chromosomal linkage groups between *T. freemani* and *T. castaneum* is presented in Table S4, where each Tfree1.0 chromosome is shown to be larger than the corresponding Tcas5.2 chromosome, ultimately resulting in a 125.9 Mb longer assembled genomic sequence for *T. freemani*.

### 2.3. Gene Annotation

**Figure 3:**
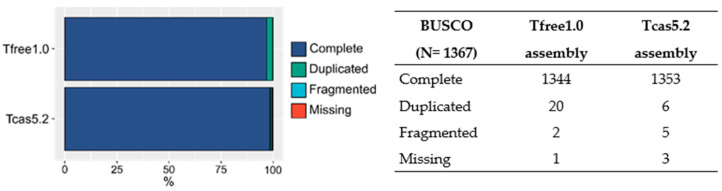
Gene completeness assessment using BUSCO analysis of *T. freemani* Tfree1.0 and *T. castaneum* Tcas5.2 assembly on all linkage groups and unplaced contigs in both species. Comparison of complete and single-copy, complete and duplicated, fragmented and missing BUSCO genes expressed in percentages (**left**) and absolute gene numbers (**right**).

In order to assess the quality of the Tfree1.0 assembly, BUSCO analysis with insecta_odb10 database was performed on all linkages groups together with 11 unplaced contigs and compared to the results of the same analysis for *T. castaneum* Tcas5.2 assembly. Gene completeness of Tfree1.0 was evaluated on a total of 1367 insect universal genes and showed that only one gene is missing, with 98.3% of them being present in complete and single-copy states and 1.5% in duplicated states (Figure 3). In comparison to Tcas5.2, Tfree1.0 showed slightly fewer fragmented or missing BUSCOs, with several more genes found in duplicated states. Nevertheless, overall levels of complete BUSCO genes are similar, indicating comparable gene completeness between the two assemblies.

Further, by taking advantage of a suitable quality of existing gene data from the closely related species, we annotated the genes on the Tfree1.0 genome assembly using the *T. castaneum* gene database. This database comprises predicted genes from two automated annotation pipelines and four ab initio prediction programs [10], and it was later reannotated and improved by evidence from RNA-seq [11]. We mapped *T. castaneum* genes to Tfree1.0 assembly using the Liftoff tool [25]. Of all 14,467 available genes present in the *T. castaneum* assembly, including genes found in unplaced contigs, 13,845 genes (95.7%) were successfully found in the Tfree1.0 assembly (Table 3). Similar is true for well-predicted mRNA, exons, and CDS regions, as there are 87–97% of them shared between the two species.

**Figure 4:**
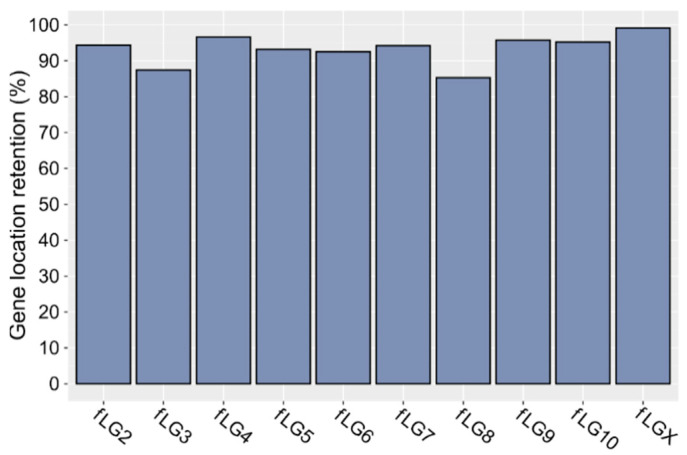
Retention of gene location between Tfree1.0 and Tcas5.2 assemblies. The bars show the percentage of *T. freemani* genes lifted from Tcas5.2 assembly that in Tfree1.0 retained location on a specific *f*LG chromosome corresponding to a specific LG chromosome in the Tcas5.2 assembly.

Next, the global localization of genes and their positioning in the two species were investigated. Gene coordinate comparison revealed that most of them (92.7%) are retained in Tfree1.0 in the same linkage group/chromosome as found in the referent *T. castaneum* assembly (Figure 4). The highest degree of gene position change can be seen on *f*LG3 and *f*LG8 (up to 15%), which can probably be mostly attributed to mapping imperfections of RagTag and Liftoff, but to some extent, it might also be a consequence of true translocation events.

### 2.4. Repeat Annotation

**Figure 5:**
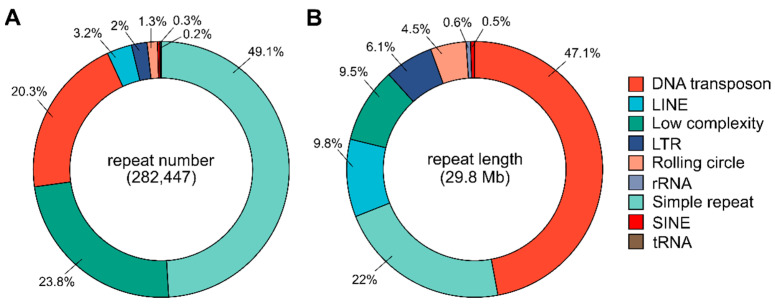
Composition of repeat elements in the *T. freemani* Tfree1.0 genome assembly. (**A**) The proportion of repeat classes in the total number of annotated repeats. (**B**) The proportion of repeat classes in the cumulative length of annotated repeats. Values are obtained by RepeatMasker annotation analysis and are shown in Table S5.

Repeat elements were annotated on the *T. freemani* Tfree1.0 assembly using RepeatMasker [26] and the database of well-curated reference repeats from Repbase [27]. Repeat elements occupy 11.1% or 29.8 Mb of the Tfree1.0 assembly (Table S5). By the number of repeats, they mostly comprise simple repeats, low-complexity regions, and DNA transposons (Figure 5A). The high abundance of simple repeats and low-complexity regions can be explained by the high AT-content of the genome (68%, Table 2), as there is an increased likelihood for them to be recognized by RepeatMasker purely due to the probabilities based on the nucleotide composition of the genome. However, when the total length is considered, DNA transposons make up almost half (47.1% or 14.1 Mb) of all annotated repeat elements (Figure 5B). In addition, significant contributors are LINE and LTR elements that occupy larger areas of 2.9 Mb and 1.8 Mb, respectively (Table S5).

**Figure 6:**
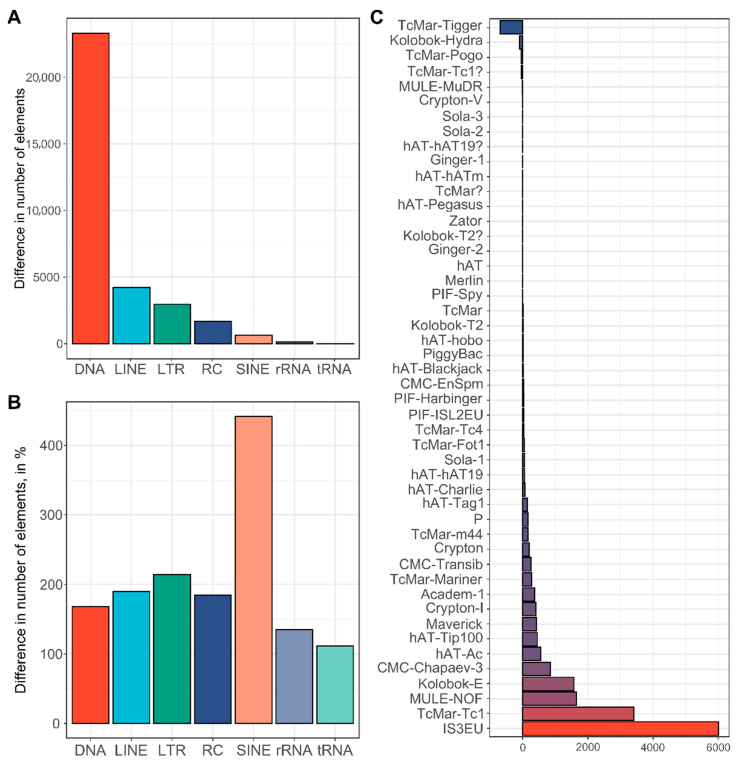
Distribution and evaluation of interspersed elements in the Tfree1.0 assembly in comparison to the Tcas5.2 assembly. (**A**) Profile of annotated interspersed elements in Tfree1.0 expressed as the difference in absolute number of elements annotated in Tfree1.0 and Tcas5.2. (**B**) Difference in number of interspersed elements in Tfree1.0 compared to Tcas5.2, expressed in percentages. (**C**) Graph of DNA transposon subclasses in Tfree1.0 plotted as increase/decrease in absolute number of elements compared to same elements present in Tcas5.2 (based on data in Table S6). The analyses were performed on the assembled linkage groups (*T. freemani f*LGs and *T. castaneum* LGs) and the unplaced contigs.

When the Tfree1.0 and Tcas5.2 assemblies are compared, an increase in representation across all interspersed repetitive categories is observable (Figure 6A, Table S5). Together with a large number of DNA transposons annotated in Tfree1.0, SINE elements show more than a 4-fold increase compared to the Tcas5.2 assembly (Figure 6B). In addition, there is a noticeable positive difference in the numbers of LTR, LINE, and rolling circle elements (Figure 6B). In order to elucidate the distribution of DNA transposons (Class II transposable elements) as major interspersed constituents of the *T. freemani* genome assembly, we performed a more in-depth comparison of specific DNA transposon subclasses and compared them between species (Figure 6C). All of the DNA transposons present in Tcas5.2 were detected in Tfree1.0 but with differences in copy number. We found that the TcMar-Tigger family is the only family significantly underrepresented in Tfree1.0 compared to Tcas5.2 (Figure 6C). Multiple families were enriched, and the most significant difference was observed for IS3EU and TcMar-Tc1 DNA transposons, which showed an increase of more than 6000 and 3400 elements in the Tfree1.0 assembly, respectively (Figure 6C, Table S6).

**Figure 7:**
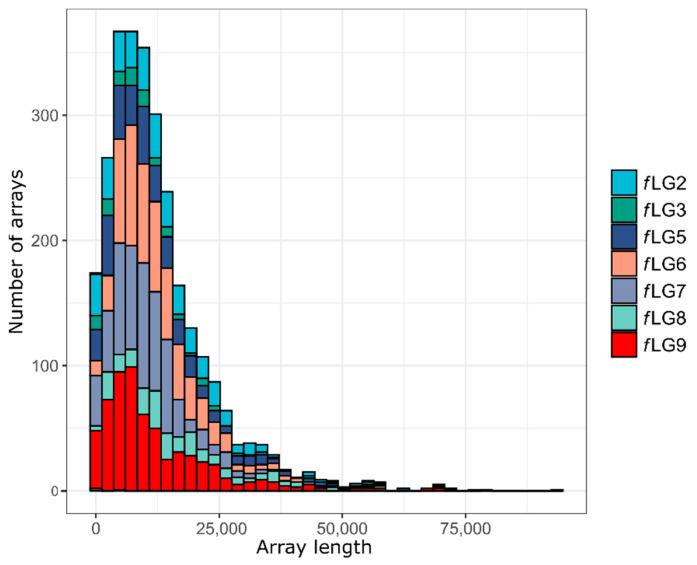
Distribution histogram of TFREE satellite DNA arrays in the *T. freemani* genome assembly Tfree1.0. Total of 2766 arrays of the TFREE satellite DNA in the assembled *T. freemani* genome are shown as they exceeded the defined stretch of five consecutive monomers used as a tandem organization criterion (Table S7). TFREE arrays have not been successfully assembled for three linkage groups (*f*LG4, *f*LG10, and *f*LGX) and thus are not visualized on the plot.

In addition to interspersed repeats, we investigated tandemly repeated, highly abundant satellite DNA TFREE that was previously described as the major satellite DNA in *T. freemani* [19]. TFREE repeat with a monomer of 166 bp was annotated in the Tfree1.0 genome assembly using megablastr, and was found to comprise 36 Mb or 13.4% of the assembled part of the genome (Table S7). The satellite DNA TFREE is organized mostly in the form of long homogeneous stretches of tandemly repeated units as visualized by dot plot (Figure 2B) and by the size distribution of TFREE arrays (Figure 7). It can be observed that half of the TFREE arrays are longer than 10 kb (Figure 7) with a maximal array length of 93.7 kb (Table S7), altogether representing huge repetitive regions.

**Figure 8:**
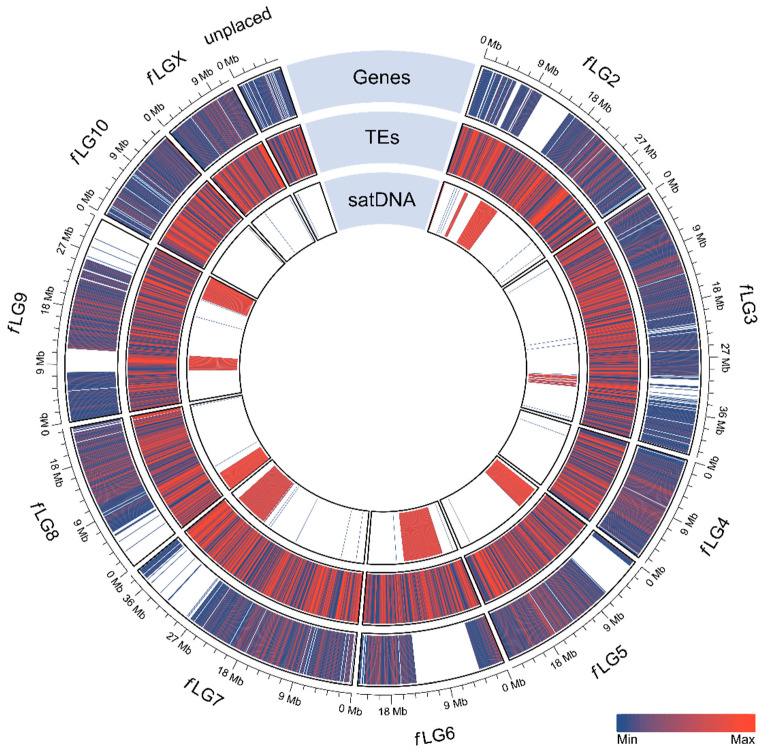
Circular visualization of the *T. freemani* genome assembly Tfree1.0. The assembly was produced by hifiasm and orientated into ten linkage groups (*f*LGs) by using RagTag and the *T. castaneum* genome assembly Tcas5.2 as a reference. The tracks represent genes, transposable elements (TEs), and the major satellite DNA (satDNA) distributed along ten chromosomal linkage groups (*f*LGs) and the unplaced contigs. Density of annotation distribution is color-coded with blue representing areas with less and red representing areas with more element-rich regions. Visualization was performed with circlize package implemented in R.

To disclose the global assembly structure, we visualized the position of tandemly repeated sequences together with interspersed elements and coding sequences on a single circular plot (Figure 8). In the Tfree1.0 assembly, several linkage groups (*f*LG4, *f*LG10, *f*LGX) are deprived of major satellite DNA blocks (Figure 8), and we assume that this could be due to assembly and contig orientation limits. It is possible that these *f*LGs are acrocentric and end in highly abundant tandem repeats, lacking unique genetic segments onto which either the assembler or the contig orientation algorithms can map to. This is also supported by the high major satDNA content in hifiasm contigs comprising 35.8% (Table S7) that is cut down to 13.4% in the final Tfree1.0 assembly, most probably due to array shrinkage and potential exclusion in acrocentric regions. Next, from the genome representation plot, it is visible that the genes are distributed along the length of all chromosomes with large gene-poor gaps that correspond to extremely satellite DNA-rich regions (Figure 8). On the other hand, transposable elements show a more scattered and uniform distribution pattern throughout the whole genome, revealing a larger tendency than genes to invade regions of long satellite arrays. Lastly, the unplaced contigs are not characterized by the presence of the TFREE satellite DNA (Figure 8). Instead, they are highly enriched in transposable elements and genes and most likely represent true genomic regions that could not be arranged due to high sequence divergence or large intra-chromosome events such as translocations or inversions.

### 2.5. Mitochondrial DNA

**Figure 9:**
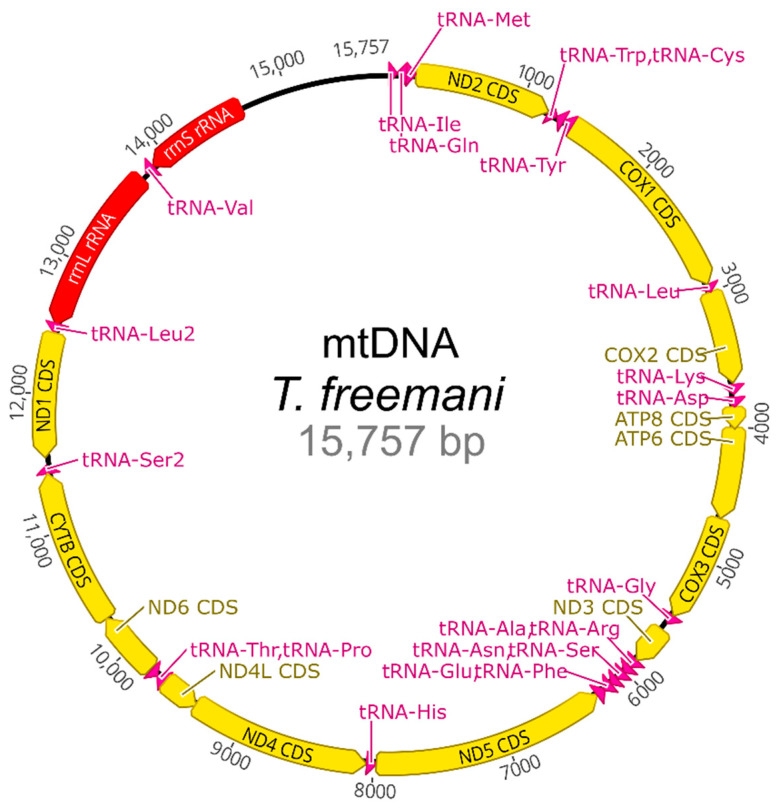
Circular map of the mitochondrial genome of *T. freemani*. Genes are labeled by standard abbreviations, and the direction of gene transcription is indicated by arrow orientation. Protein-coding genes are shown in yellow, ribosomal RNA genes in red, transfer RNA genes in magenta, and the control region in black.

Together with the nuclear genome, we assembled the *T. freemani* mitochondrial genome. The mitogenome was obtained from hifiasm assembled contigs by MitoFinder pipeline using the mitochondrial DNA (mtDNA) of closely related *T. castaneum* as a reference. The mtDNA of *T. freemani* was found present in one contig. The complete mitochondrial genome is 15,757 bp long and contains 13 protein-coding genes, 22 transfer RNA (tRNA) genes, 2 ribosomal RNA (rRNA) genes, and an AT-rich control region (Figure 9).

In order to compare the conservation of mitogenome between *T. freemani* and *T. castaneum*, their gene content was evaluated. In addition to their similar mtDNA sizes, the two species share collinearity of all 37 mitochondrial genes. The genes showed a high degree of conservation, with most of the genes having pairwise similarity scores above 85% (Table S8). As mitochondrial genomes are valuable sources of sequence data for phylogenetic analysis, we compared *T. freemani* mtDNA to 10 coleopteran mtDNAs to test its applicability at different taxonomic levels. The ML phylogenetic analysis recognized *T. freemani* and *T. castaneum* mtDNAs as the most closely related, and it placed the four tested *Tribolium* mitogenomes into a separate group supported by a 100% bootstrap value (Figure S3), perfectly reflecting their intragenomic species-groups distribution as suggested by Hinton [12]. The ML analysis also clustered tenebrionid mtDNAs into one group, separating them from the mtDNAs of other coleopteran superfamilies (Figure S3), thus confirming mtDNA relevance for phylogenomics of close related (congeneric level) and distant species (suborder level).

## 3. Discussion

In this work, the genome of the flour beetle *T. freemani* has been sequenced and assembled. Our motivation was to provide a genome sequence of the species that is the most closely related to the coleopteran representative species *T. castaneum*, the important food pest, and the second most popular model insect after *Drosophila* [11]. Many sequencing consortia emphasize the necessity of sequencing not only representative species but also their near relatives because various genome traits, including the correlatives of species boundaries, can only be revealed through the comparison of close sister species [28].

To provide a high-quality genomic sequence, we opted for highly accurate PacBio HiFi sequencing. The analysis of k-mer frequencies in 23.8 Gb of sequencing data estimated the approximate genome size of *T. freemani* to be around 320 Mb. This genome size estimation is higher than the previous estimation of 230 Mb, calculated from the densitometric measurements of nuclear content of spermatids, which showed significant individual heterogeneity [16]. Although computational predictions of genome size performed with different programs showed variability, k-mer analyses estimated that approximately one-third of the *T. freemani* genome is made of repeat sequences.

The presumed highly repetitive content of the *T. freemani* genome had the largest impact in directing our choice of an assembly pipeline. In spite of significant progress in sequencing technologies and assembly methods, repetitive sequences such as tandem repeat arrays and segmental duplications still pose a major challenge for creating accurate high-resolution or gapless genomic maps [29], hindering ab initio genome assembling in particular. It has been shown that a reference-guided de novo assembly approach facilitates assembling but also outperforms the corresponding de novo assembly strategy without a reference genome [30]. Reference-assisted scaffolding avenues have been used successfully in generating many genome assemblies, including those of insects [31]. Therefore, in our endeavor to decipher the *T. freemani* genomic sequence, we decided to use the latest version of the high-quality *T. castaneum* genome assembly Tcas5.2 [11] as a reference. Aware of the large share of tandemly repeated sequences in both genomes [18,19] and the extent to which they can obstruct accurate assembling, we chose a gene-focused assembly strategy favoring gene-enriched contigs. With this approach, we assembled 262 Mb of the genomic sequence by anchoring 99 highly contiguous *T. freemani* contigs into 10 pseudomolecules/linkage groups. Based on the BUSCO assessment using insect universal orthologous genes as a reference, the completeness of the *T. freemani* assembly Tfree1.0 was estimated to be 99.8%, indicating a high-quality assembled genome comparable in quality to other available insect genome assemblies [7]. The 10 *T. freemani* pseudomolecules (*f*LGX-*f*LG10) correspond to the 10 *T. castaneum* chromosomes (9 autosomal chromosomes and the X), indicating a chromosome-level assembly based on their continuity.

The comparison of the *T. freemani* and *T. castaneum* genome assemblies showed that the two sibling species are very similar in their coding sequence. We successfully annotated 95.7% of the *T. castaneum* genes in the *T. freemani* assembly, and among orthologous genes, 92.7% keep their position on the corresponding chromosomes. The observed differences in the number of annotated genes coincide with the cumulative length of the coding sequences, which is 40.8 Mb in Tfree1.0 and 44.9 Mb in Tcas5.2. We nevertheless hypothesize that the smaller number of genes annotated in the Tfree1.0 assembly is not necessarily due to their effective loss in the *T. freemani* genome. It could be that some genes in the Tfree1.0 assembly were not recognized in the Liftoff analysis because they diverged from *T. castaneum* genes to an extent below the sensitivity of the algorithm. It is also possible that a smaller number of genes is concealed in highly repetitive regions/contigs that were not included in the assembly, but mapping of uniquely mappable features within the unassembled contigs indicates that the number of “discarded” genes cannot be large.

Regarding the non-coding part of the genome, repetitive DNA was estimated to comprise >30% of *T. castaneum* and *T. freemani* genomes ([32], this work). Although neither *T. castaneum* Tcas5.2 nor *T. freemani* Tfree1.0 assemblies were completed in their repetitive regions, the available data allowed us to compare repetitive elements within the assembled regions. Inspection of individual repetitive elements disclosed distinctions between the two species. The same categories of interspersed repeats are found in both assemblies in similar proportions, but the interspersed elements occupy a 17 Mb longer sequence in the Tfree1.0 assembly, suggesting their proliferation in the *T. freemani* genome. However, this assumption should be taken with caution because the assemblies of the two species did not derive from the same type of sequencing data, and it is possible that HiFi reads allowed longer repetitive regions to be assembled in Tfree1.0, thus allowing more elements to be annotated. Among the interspersed repeats present in the Tfree1.0 assembly, DNA transposons show the largest difference in number compared to Tcas5.2, especially for IS3EU and TcMar-Tc1 elements. Interestingly, TcMar-Tc1 transposons have been found highly expanded in the genome of the nematode *Caenorhabditis inopinata*, a recently discovered sibling species of *Caenorhabditis elegans*, and it was assumed that they could be the main driver for the genome size differences between *Caenorhabditis* species [33]. Although interspersed repeats make up a significant proportion of the *T. freemani* genome, tandem repeats undoubtedly contribute the most to the genome size. The most dominant sequence in the *T. freemani* genome is TFREE, the major satellite DNA that was identified 30 years ago [19]. The analyses of eight species of the genus *Tribolium* have shown that *Tribolium* genomes tend to accumulate large amounts of satellite DNAs, which can occupy up to 60% of the genome [18,19,34,35,36,37,38], and most of them, except for *T. audax*/*T. madens* satellite DNAs are species-specific. In terms of nucleotide sequence, TFREE satellite DNA is not related to the *T. castaneum* major satellite DNA TCAST, but both satellite DNAs form large blocks of heterochromatin in (peri)centromeric regions [18,19,39]. Due to tandem organization, these sequences remain largely excluded from the assembled parts of the genome, and TCAST composes only 0.3% of the assembled *T. castaneum* genome [11,32,39]. In the Tfree1.0 assembly, we annotated long arrays of TFREE tandem repeats that cumulatively comprise 36 Mb, making 13.4% of the assembled sequence. We assume that the remaining 50 Mb of the estimated missing genome sequence could be primarily made of TFREE. The fact that in situ hybridization detected large TFREE arrays on all *T. freemani* chromosomes [19], while we failed to assemble them on three chromosomes, due to their acrocentric architecture and highly repetitive content, speaks in favor of our assumption. Even though Tfree1.0 assembly does not provide the comprehensive assembly of the major satellite DNA, the estimated 80–90 Mb of TFREE repeats in *T. freemani* versus approximate 30–40 Mb of TCAST repeats in *T. castaneum* genome unequivocally points to the satellite DNA as the most substantial quantitative difference in genomic sequence between the two sibling species.

*T. freemani* and *T. castaneum* diverged approximately 14 Mya [40], but they still can hybridize [13]. Given the unexplored geographical distribution of *T. freemani*, it is not known whether the two species meet in nature, but in laboratory conditions, they produce sterile F1 hybrid progeny in reciprocal crosses, revealing postzygotic reproductive isolation. It is tempting to speculate that the prodigious disparity in major satellite DNAs and the heterochromatin in the (peri)centromeric regions they build in *T. freemani* and *T. castaneum* might lead to the reproductive isolation of the two species. The impact of satellite DNA divergence on interspecies hybrids’ lethality and sterility has already been reported for animal sibling species. Satellite DNA-based variations of heterochromatin impact chromosome segregation and ultimately result in hybrid lethality of *Drosophila melanogaster* and *Drosophila simulans* sibling crosses [41], and could also play a role in speciation of mosquitoes from the *Anopheles gambiae* group, whose sibling species produce sterile F1 hybrids [42]. On the other hand, hybrid dysfunction can be caused by large-scale alternations in gene expressions, as was shown for sterile mice hybrids between *Mus musculus musculus* and *Mus musculus domesticus* subspecies [43], or for F1 hybrids of the nematode sibling species *Caenorhabditis briggsae* and *Caenorhabditis nigoni* [44]. From the “zoomed-out” perspective, highly divergent satellite DNAs and a core set of genes, which *T. freemani* and *T. castaneum* appear to share, represent the structural and functional antipodes of their genomes. An extensive and comprehensive future research of both coding and non-coding DNAs will be needed to address an appealing question of the genetic basis of *T. freemani*-*T. castaneum* postmating incompatibilities, and their hybrids certainly could serve as a worthy model for studying postzygotic reproductive barriers between sibling species.

Along with the nuclear genome, we also assembled the *T. freemani* mitochondrial genome. With conserved order and orientation of 37 genes that it encodes, the *T. freemani* mitogenome is consistent with an ancestral insect mitochondrial genome [45]. Phylogenetic analysis on a limited number of taxa showed that among related species, the *T. freemani* mitogenome is most similar to that of *T. castaneum*, also perfectly reflecting taxonomic relationships between tested species at the suborder level. In addition to their most prevalent usage for molecular systematics at different taxonomic scales, mitochondrial genomes of some insects have been reported to be related to insecticide resistance. For example, changes in sequence and expression of some mitochondrial genes in the malaria vector *Anopheles sinensis* appeared to be associated with resistance to the pyrethroid insecticide [46], while changes in the expression level of mitochondrial-encoded genes in *Drosophila melanogaster* 91-R strain are linked to DDT resistance [47]. Differential expression of the genes related to mitochondrial functions was also found in the *T. castaneum* population resistant to phosphine, a fumigant used for stored products treatment to control pests [48]. Although the ecological range of *T. freemani* is unknown and thus assumed to be limited, its easy rearing on the wheat medium and other food commodities, under the conditions optimal for *T. castaneum*, suggests that *T. freemani* has a great potential to be a serious stored product pest [13]. Being a closest sibling of one of the most important worldwide pest insects, *T. freemani* could serve as a comparative model for studying insecticide resistance mechanisms and potentially be applied in the development of novel pest management approaches.

In conclusion, here we provided de novo assembly of the remarkably repetitive genome of the flour beetle *T. freemani*. Repetitive sequences are the most severe obstructers of gapless assemblies, and very often, it is not possible to assemble them correctly, even with great effort [49]. Our Tfree1.0 assembly certainly needs to be improved and completed in repeated regions; however, by reference-guided gene-oriented strategy, we assembled the genomic sequence approaching the chromosomal level. While waiting for the follow-up attempts to resolve those highly repetitive stretches, we release the first, high-quality version of the *T. freemani* assembly together with its mitogenome for the research community to exploit this background information for further discoveries.

## 4. Materials and Methods

### 4.1. Insect Material

The initial stock of the flour beetle *T. freemani* was obtained from USDA-ARS (Manhattan, KS, USA) in 2015 and maintained as a laboratory culture. The insects were reared in the whole wheat flour at 27 °C and 55% humidity in the dark, being sub-cultured every four weeks.

### 4.2. DNA Extraction and PacBio HiFi Sequencing

Genomic DNA was isolated from 24 snap-frozen pooled male and female larvae using the Qiagen Genomic tip 100 kit (Qiagen, Germantown, MD, USA). DNA isolation and library preparation using SMRTbell Express Template Prep Kit 2.0 (Pacific Biosciences, Menlo Park, CA, USA) were performed by the sequencing provider. PacBio HiFi sequencing was performed at DNA Sequencing Centre (DNASC) at Brigham Young University (Provo, UT, USA) using the Sequel II System machine. The sequencing resulted in 1,617,087 HiFi reads with a total length of 23.8 Mb. The quality of HiFi reads was assessed with FastQC, and no remaining adapters or specific overrepresented sequences were found.

### 4.3. Genome Size Estimation

Genome size estimation was performed using three different publicly available programs: GenomeScope [20], findGSE [21] and CovEST [22]. The algorithms have been developed for estimating genome sizes from k-mer occurrences using different mathematical models from Illumina short reads sequencing data; however, due to the low error rate of PacBio HiFi technology, all of the models are also applicable to PacBio HiFi reads [50]. All three coverage estimates required the previous creation of a histogram file with the jellyfish program [51] using the following command:

jellyfish count -C -m xx -s 4000000000 -t 16 reads.fasta -o reads.jf

where the “-m” flag was changed according to the different required k-mer size used in the subsequent prediction. After the creation of the jellyfish count file, a histogram file was created using the jellyfish “histo” module with “xx” representing the k-mer size used:

jellyfish histo -t 16 reads.jf > reads_mxx.histo

Afterward, the programs for genome size estimation were run by applying the specified commands for each of the programs. GenomeScope was run with the following command:

Rscript genomescope.R reads.histo xx 15000./genoscope_output/

The findGSE program was run from R using the CRAN deposited library and the following function call:

findGSE(histo=“reads_mxx.histo”, sizek=xx, outdir=“./”)

Finally, CovEst was run using the following command:

covest -m ‘repeats’ -k xx -r 15000 reads_mxx.histo > covest_repeats_mxx.txt

where “xx” represents the k-mer size used, and the number ”15000” represents the N50 of the reads.

### 4.4. Reference Sequence

The reference assembly used in this work was the *T. castaneum* genome assembly Tcas5.2 [11], available in the NCBI genome database under the accession number GCA_000002335.3. The Tcas5.2 assembly consists of contigs that have been anchored to ten pseudomolecules (accessions CM000276-CM000285), representing linkage groups (LG) of nine autosomes (LG2-LG10) and the X chromosome (LGX = LG1), with the remaining sequence represented as unplaced scaffolds and unplaced singletons, including the unassembled y chromosome. The assembly also includes the newly revised and curated gene set OGS3 [11], which is used for *T. freemani* gene annotation on hifiasm contigs and the final *T. freemani* assembly.

### 4.5. T. castaneum Assembly Gap Filling

In order to successfully map and orient *T. freemani* hifiasm contigs, we decided first to fill in gaps present in the *T. castaneum* reference assembly Tcas5.2 with as much *T. freemani* genomic information as possible. Using the same HiFi reads as for hifiasm assembly, we performed gap filling with the TGS-GapCloser program, as described in the reference manual [52], with minimap2 [53] parameters best suited for mapping HiFi reads onto the Tcas5.2 assembly, allowing high sequence divergence because of differences we expected between the two species. The tool was run with the following command:

TGS-GapCloser.sh --scaff tcast52_assembly.fasta --reads t_free_pacbio.fasta --output gap_filled_freemani --ne --tgstype pb --minmap_arg ‘-x asm20’ > pipe.log 2>pipe.errAfter

### 4.6. GenomeAassembly

The assembly of *T. freemani* HiFi reads into contigs was performed with hifiasm [23] using the options specified in the reference manual and applicable for highly repetitive genomes:

hifiasm -N 200 -a 6 -o freemani_assembly -t 64 reads.fastq

Due to the fact that BUSCO showed a high level of gene completeness (Figure 3) and because hifiasm performs six rounds of error correction, we deemed that it was not necessary to perform genome polishing since no new information would be included in the assembly. The orientation of contigs into chromosome-level scaffolds was performed using the RagTag algorithm [24]. In short, RagTag first uses minimap2 algorithm to find optimal positions and orientations of contigs and then concatenates all the placed contigs into single chromosome-level units. The RagTag algorithm was called with filtered contigs and the gap-filled *T. castaneum* assembly using the following command:

ragtag.py scaffold gap_filled_freemani_2.fasta filtered_tigs.fasta -r -o./scafolding_6 -C -w -f 200000

where “-r” is the option to imply gap sizes in order to produce an assembly of the highest similarity. The dot plot graphs were created using the dotPlotly (https://github.com/tpoorten/dotPlotly) algorithm (accessed on 15 January 2022).

### 4.7. GeneAanalysis and Liftoff

The *T. castaneum* gene set (from GCF_000002335.3_Tcas5.2_genomic.gff) was lifted onto the contigs to filter out only those contigs that carry genetic information that have unique genetic mapping. The lifting was performed using the Liftoff program [25] and the following line:

liftoff -g tcast_52_annots.gff3 -m./liftoff/minimap2/minimap2 tcast_assembly.fasta contigs.fasta -o lifted _genes_to_contigs.gff3 -p 16 -copies

With the “copies” flag, we have allowed multiple copies of the same feature to be mapped in case of uncollapsed contigs and/or duplication. Here, the term “feature” represents any gene, exon, CDS, mRNA, transcript, lnc_RNA, primary_transcript, miRNA, or pseudogene annotation, as annotated in the *T. castaneum* official gene set OGS3. As the specified features have a non-repetitive qualification in the T.cas5.2 assembly, we named them uniquely mappable features (abbreviated, UMFs). After lifting the UMFs, we selected only those hifiasm contigs that had more than 10 UMFs and used them for the subsequent assembly. In the same manner, we performed final gene lifting from *T. castaneum* to the *T. freemani* assembly produced by RagTag.

Benchmarking Universal Single-Copy Orthologs (BUSCO) is a highly used measure for quantitative assessment of genome assembly and annotation completeness based on evolutionarily informed expectations of gene content for the genome of interest [54]. All BUSCO analyses were performed with the BUSCO v5.0.0. module on the Galaxy web platform (usegalaxy.org, accessed on 10 May 2022) using “insectaodb10” as the gene set marking full completion.

### 4.8. Repeat Analyses

RepeatMasker is a widely used tool for finding and masking different repeat elements within a given target sequence [26]. RepeatMasker is used here in order to obtain the GFF/GTF formatted data with the position and orientation of different classified RepBase repeat elements, from which quantity, size, and distribution of different elements were examined. All masking was performed on the Galaxy server using the RepBase RELEASE 20181026 and RepeatMasker (4.0.9_p2). All of the repeat content and sequence analyses were performed on the GFF files. Annotation and discovery of the major satellite DNA repeats within the *T. freemani* assembly were performed using the NCBI’s BLAST algorithm and the interface to the R programming language package metablastr [55]. As a query, the TFREE satellite monomer sequence (GenBank entry X58539.1) was used, and the repeat was discovered if the BLAST algorithm resulted in a query coverage and percent identity >70% for the satellite DNA sequence.

### 4.9. Mitochondrial DNA

The *T. freemani* mitochondrial DNA (mtDNA) sequence was extracted from hifiasm assembled contigs instead from reads, as showed in [56], for increased speed and accuracy using the MitoFinder algorithm, settings of which have been handled and run with MitoHifi [57]. The *T. freemani* mtDNA was found present in three highly conserved consecutive copies (99.99% identity) within a 51.7 kb long contig ptg000244l. The mtDNA of closely related *T. castaneum* [58] was used as the reference point for discovery. The obtained *T. freemani* mtDNA was compared to whole mtDNAs of 11 insect species, whose sequences were retrieved from the NCBI GenBank database as follows: *T. castaneum* (NC003081.2), *Tribolium audax* (KJ752724.2); *Tribolium confusum* (KP420018.1), *Tenebrio molitor* (KP994554.1), *Asbolus verrucosus* (KP698408.1), *Gonocephalum kochi* (MW822744.1), *Ulomoides dermestoides* (KM046492.1), *Rhyzopertha dominica* (MW020612.1), *Platisus zelandicus* (MK614519.1), *Dorcasomus pinheyi* (MN447435.1), and *Drosophila melanogaster* (NC_024511.2). The sequences were aligned using the MUSCLE algorithm [59] with the maximum number of 4 iterations with kmer4_6 distance measure, UPGMB clustering, and CLUSTALW sequence weighting scheme. The maximum likelihood (ML) tree was reconstructed based on the Hasegawa–Kishino–Yano model using MEGA 11.0.10 [60]. Statistical testing of the robustness of the tree topology was performed by bootstrap resampling of 500 replications.

## Figures and Tables

**Table 1:** Summary of *T. freemani* genome size and repeat ratio estimation. Three different programs (GenomeScope, findGSE, CovEST RE) were used with varying k-mer sizes.

Algorithm	Genome Size Prediction (Mb)				Estimated Repeat Ratio (%)			
	k-mer Size			Average	k-mer Size			Average
	21	31	41		21	31	41	
GenomeScope	189.6	202.2	212.8	201.5	30.4	28.4	28	28.9
findGSE	225.6	240.4	255.7	240.6	33.9	32.4	32.4	32.9
CovEST RE	238.7	305.5	412.3	318.8				

**Table 2:** Statistics of raw PacBio HiFi data, contigs obtained using hifiasm assembler, and contigs used for assembly with RagTag algorithm producing the assembly Tfree1.0.

Raw PacBio Data		Hifiasm Output		Tfree1.0	
**Number of** **reads**	1,617,087	**Number of contigs**	679	**Number of contigs**	110
**Total length** **(bp)**	23,796,436,578	**Total length (bp)**	465,826,150	**Total length** **(bp)**	269,018,543
**Number of** **reads <5000 bp**	301	**Min contig length (bp)**	13,227	**Min contig** **length (bp)**	22,590
**Largest read** **(bp)**	42,982	**Max contig length (bp)**	23,376,958	**Max contig length (bp)**	23,376,958
**GC (%)**	32	**GC (%)**	31.52	**GC (%)**	32.53
**N50**	14,965	**N50**	5,522,289	**N50**	8,487,211
**N90**	11,545	**N90**	345,137	**N90**	1,794,767
**L50**	675,343	**L50**	23	**L50**	10
**L90**	1,394,918	**L90**	160	**L90**	30

**Table 3:** Comparison of assembly annotations between the reference *T. castaneum* Tcas5.2 assembly and features lifted to the *T. freemani* Tfree 1.0 assembly.

Genomic Feature	Tcas5.2	Tfree1.0	Retained Data (%)
**Gene**	14,467	13,845	95.70
**mRNA**	22,598	21,936	97.07
**Exon**	171,320	149,045	87.00
**CDS**	22,611	21,827	96.53
**Transcript**	317	289	91.17
**lncRNA**	1364	1165	85.41
**Primary transcript**	220	144	65.45
**tRNA**	247	237	95.95

## Data Availability

The *T. freemani* genome assembly Tfree1.0 has been deposited to the European Nucleotide Archive (ENA) under the BioProject accession PRJEB52307 with the assembly accession number GCA_939628115. The annotated data presented in this study are openly available in FigShare at 10.6084/m9.figshare.19682400. The annotated *T. freemani* mitochondrial DNA has been deposited in the NCBI GenBank database under the accession number ON303726.

## Supplementary Materials

The following supporting information can be downloaded at: https://www.mdpi.com/article/10.3390/ijms23115869/s1.
