# Supplementary material for: Reference-Guided De Novo Genome Assembly of the Flour Beetle Tribolium freemani
Source: Int J Mol Sci. 2022 May 24;23(11):5869. doi: 10.3390/ijms23115869 (PMC9180572; doi:10.3390/ijms23115869)
Supplement: Supplementary file 1 [file ijms-23-05869-s001.zip › Figure S1.pdf]

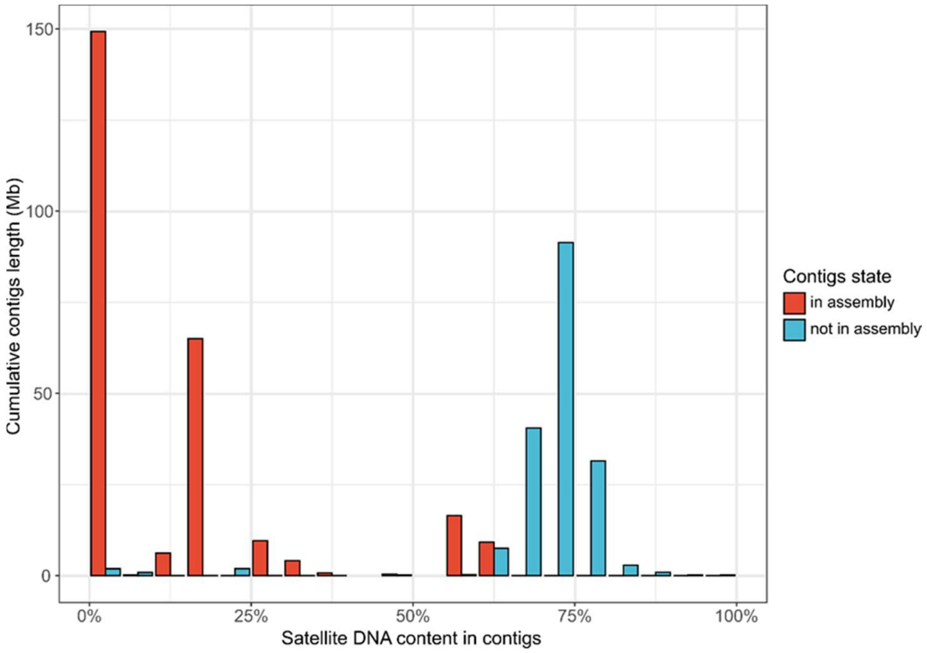

Figure S1. Histogram showing the relationship between the relative content of the satellite DNA TFREE in the 679 contigs produced by the hifiasm assembler and the cumulative length of the contigs. The 110 contigs included in the final Tfree1.0 assembly are contained within the red bars, while the blue bars contain 569 contigs not included in the Tfree1.0 assembly.
