# Supplementary material for: Reference-Guided De Novo Genome Assembly of the Flour Beetle Tribolium freemani
Source: Int J Mol Sci. 2022 May 24;23(11):5869. doi: 10.3390/ijms23115869 (PMC9180572; doi:10.3390/ijms23115869)
Supplement: Supplementary file 1 [file ijms-23-05869-s001.zip › Figure S2.pdf]

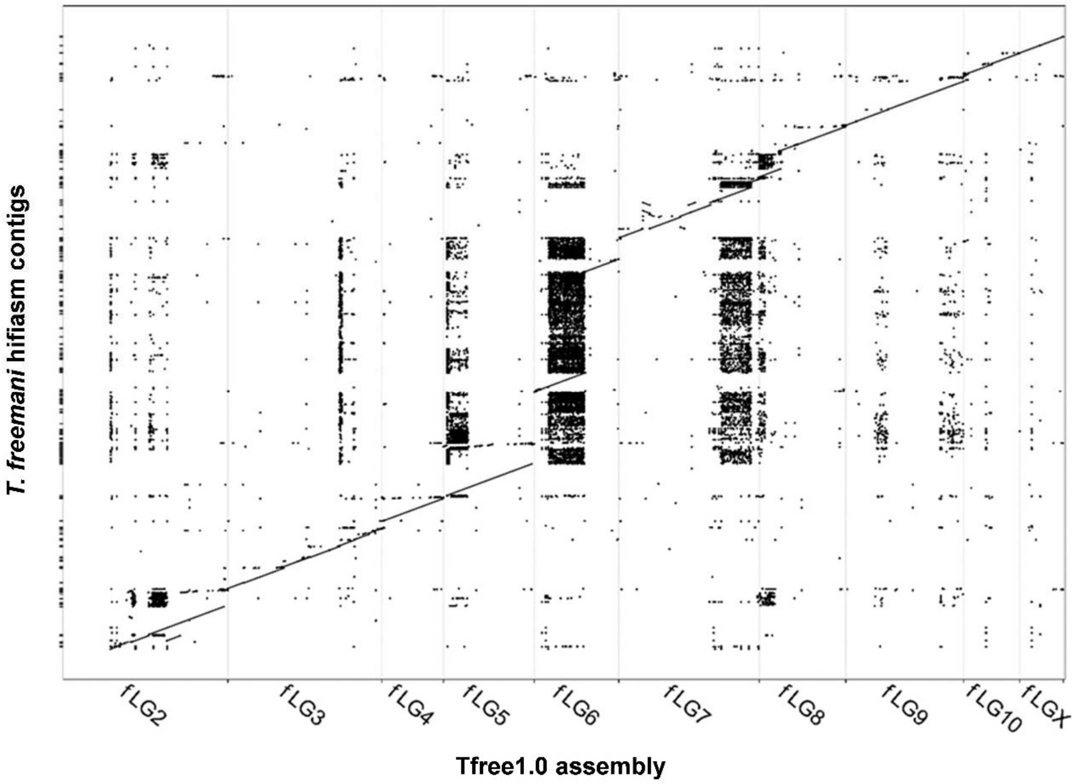

Figure S2. Dot plot analysis of 679 hifiasm output contigs mapped on the final Tfree1.0 assembly. The dot plot shows that the Tfree1.0 assembled sequence is covered along its entire length by hifiasm contings, many of which are mapped to regions of highly repetitive DNA (indicated by dark blocks).
