# Supplementary material for: Reference-Guided De Novo Genome Assembly of the Flour Beetle Tribolium freemani
Source: Int J Mol Sci. 2022 May 24;23(11):5869. doi: 10.3390/ijms23115869 (PMC9180572; doi:10.3390/ijms23115869)
Supplement: Supplementary file 1 [file ijms-23-05869-s001.zip › Figure S3.pdf]

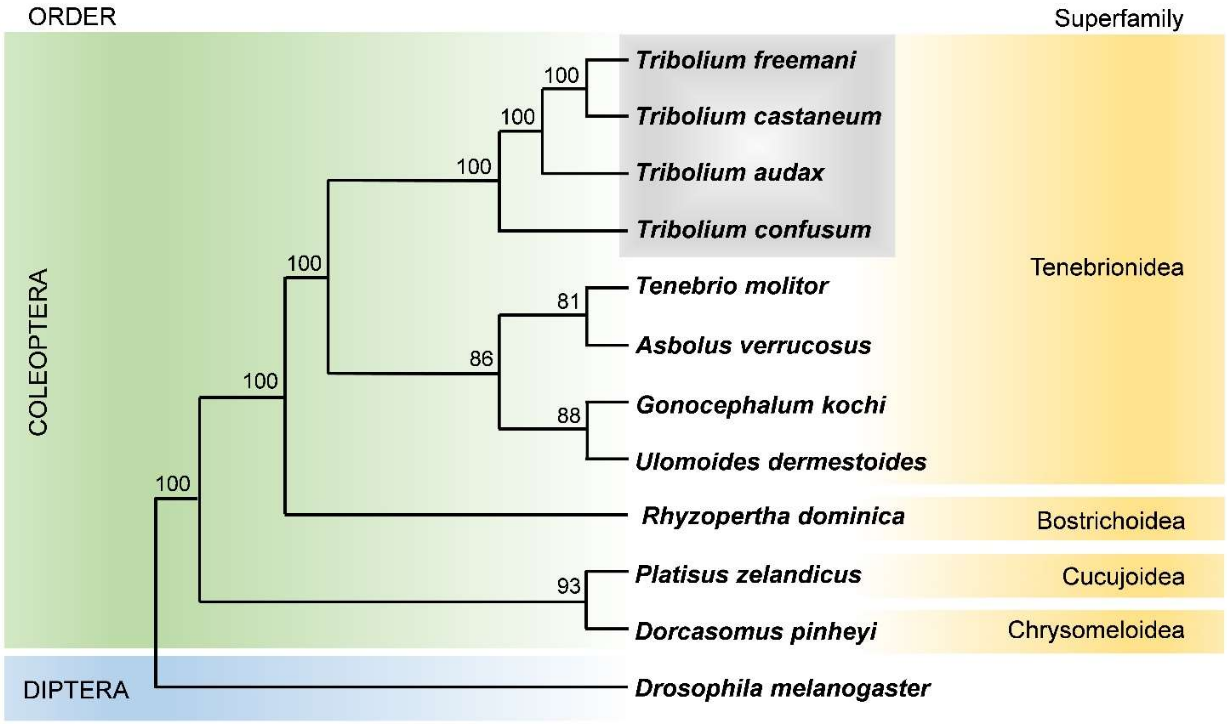

Figure S3. The Maximum Likelihood tree showing relationships of 11 coleopteran mtDNA sequences. *T. freemani* mtDNA sequence was generated in this work, while the mtDNA sequences for other species were retrieved from the NCBI GenBank database (the accession numbers are listed in Materials and Methods section). Bootstrap support values based on 500 replicates are indicated above branches, and the fruit fly *Drosophila melanogaster* was used as an outgroup.
