# Supplementary material for: Reference-Guided De Novo Genome Assembly of the Flour Beetle Tribolium freemani
Source: Int J Mol Sci. 2022 May 24;23(11):5869. doi: 10.3390/ijms23115869 (PMC9180572; doi:10.3390/ijms23115869)
Supplement: Supplementary file 1 [file ijms-23-05869-s001.zip › Table S1.pdf]

Table S1. Distribution of the major satellite DNA TFREE monomers and uniquely mappable features (UMFs) within the 679 contigs obtained with hifiasm assembler.

Hifiasm contig	Contig length (bp)	Contig status in Tfree1.0 assembly	Number of uniquely mappable features (UMFs) within the contig	Number of TFREE satellite DNA monomers within the contig	Percentage of contig length occupied by TFREE satellite DNA (%)
ptg000016l	23376958	In assembly	38044	27927	0,20
ptg000002l	20865910	In assembly	27702	27228	0,21
ptg000003l	20620739	In assembly	25327	24036	0,19
ptg000012l	16082494	In assembly	28309	1086	0,01
ptg000020l	12447313	In assembly	830	45472	0,60
ptg000008l	12424795	In assembly	20937	321	0,00
ptg000041l	10608858	In assembly	18431	197	0,00
ptg000030l	9178789	In assembly	46	37621	0,67
ptg000004l	8542712	In assembly	4245	16666	0,32
ptg000060l	8487211	In assembly	18109	50	0,00
ptg000071l	8209875	In assembly	14728	211	0,00
ptg000048l	7974590	In assembly	12880	42	0,00
ptg000006l	7917093	In assembly	7471	634	0,01
ptg000069l	7541998	In assembly	13579	175	0,00
ptg000061l	6519519	In assembly	4513	127	0,00
ptg000005l	6399338	In assembly	24398	156	0,00
ptg000319l	6193494	In assembly	8517	304	0,01
ptg000031l	5522289	In assembly	7757	143	0,00
ptg000055l	4641919	In assembly	2275	345	0,01
ptg000117l	4462153	In assembly	3855	4155	0,15
ptg000021l	4264982	In assembly	5545	35	0,00
ptg000045l	4120959	In assembly	298	8410	0,33
ptg000019l	4119783	In assembly	4936	4	0,00
ptg000009l	4069966	In assembly	12	14664	0,59
ptg000022l	3887479	In assembly	1938	44	0,00
ptg000039l	3321203	In assembly	4663	355	0,01
ptg000166l	2960622	In assembly	2153	208	0,01
ptg000052l	2165914	In assembly	337	1235	0,06
ptg000082l	2080578	In assembly	804	80	0,00
ptg000173l	1794767	In assembly	1293	107	0,01
ptg000102l	1720055	In assembly	262	1652	0,15
ptg000144l	1678416	In assembly	1015	75	0,01
ptg000107l	1561342	In assembly	386	95	0,01
ptg000046l	1531797	In assembly	816	3	0,00
ptg000150l	1241913	In assembly	240	67	0,01
ptg000103l	1183169	In assembly	134	242	0,02
ptg000095l	1153942	In assembly	109	204	0,02
ptg000036l	1042122	In assembly	1310	113	0,02
ptg000342l	1041323	In assembly	867	1818	0,29
ptg000010l	941320	In assembly	1631	37	0,01
ptg000179l	762607	In assembly	74	332	0,05
ptg000243l	743376	In assembly	20	1791	0,40
ptg000112l	709056	In assembly	231	7	0,00

ptg000311l	684097	In assembly	225	2	0,00
ptg000140l	660759	In assembly	464	0	0,00
ptg000105l	618581	In assembly	352	62	0,02
ptg000251l	600520	In assembly	295	4	0,00
ptg000215l	564234	In assembly	1423	8	0,00
ptg000194l	539326	In assembly	76	38	0,01
ptg000228l	533472	In assembly	91	0	0,00
ptg000264l	508407	In assembly	416	3	0,00
ptg000101l	460667	In assembly	22	1347	0,49
ptg000196l	422899	In assembly	313	0	0,00
ptg000148l	410420	In assembly	276	0	0,00
ptg000111l	382289	In assembly	4040	0	0,00
ptg000198l	318746	In assembly	259	6	0,01
ptg000321l	286311	In assembly	51	0	0,00
ptg000099l	275551	In assembly	61	0	0,00
ptg000200l	265695	In assembly	85	5	0,00
ptg000306l	255908	In assembly	25	5	0,00
ptg000225l	245249	In assembly	53	0	0,00
ptg000245l	228335	In assembly	62	175	0,12
ptg000162l	226747	In assembly	422	0	0,00
ptg000368l	214582	In assembly	104	0	0,00
ptg000081l	204053	In assembly	100	2	0,00
ptg000057l	196613	In assembly	272	0	0,00
ptg000378l	176107	In assembly	12	80	0,07
ptg000330l	176036	In assembly	287	0	0,00
ptg000303l	175816	In assembly	74	0	0,00
ptg000384l	173970	In assembly	23	3	0,00
ptg000341l	169908	In assembly	13	0	0,00
ptg000426l	167095	In assembly	26	0	0,00
ptg000293l	163589	In assembly	43	0	0,00
ptg000525l	162264	In assembly	169	0	0,00
ptg000413l	158778	In assembly	249	0	0,00
ptg000419l	148526	In assembly	390	0	0,00
ptg000372l	147764	In assembly	65	174	0,19
ptg000359l	139321	In assembly	36	8	0,01
ptg000461l	130433	In assembly	32	0	0,00
ptg000236l	127976	In assembly	30	0	0,00
ptg000397l	126202	In assembly	61	0	0,00
ptg000253l	124306	In assembly	123	0	0,00
ptg000444l	121480	In assembly	29	0	0,00
ptg000463l	116516	In assembly	63	0	0,00
ptg000302l	115069	In assembly	69	0	0,00
ptg000202l	109599	In assembly	131	29	0,04
ptg000399l	108752	In assembly	26	4	0,01
ptg000246l	107246	In assembly	38	0	0,00
ptg000353l	106235	In assembly	71	0	0,00
ptg000326l	97220	In assembly	363	0	0,00
ptg000266l	96573	In assembly	48	9	0,02
ptg000329l	82499	In assembly	104	0	0,00

ptg000420l	77294	In assembly	31	0	0,00
ptg000249l	75271	In assembly	12	0	0,00
ptg000503l	75193	In assembly	25	0	0,00
ptg000282l	71981	In assembly	16	0	0,00
ptg000083l	69240	In assembly	17	0	0,00
ptg000232l	61143	In assembly	79	9	0,05
ptg000531l	52587	In assembly	25	0	0,00
ptg000424l	52008	In assembly	16	0	0,00
ptg000570l	51150	In assembly	25	0	0,00
ptg000328l	49805	In assembly	12	0	0,00
ptg000389l	49519	In assembly	26	0	0,00
ptg000437l	49136	In assembly	47	0	0,00
ptg000556l	43264	In assembly	29	0	0,00
ptg000396l	41312	In assembly	17	0	0,00
ptg000406l	38852	In assembly	37	0	0,00
ptg000429l	33086	In assembly	72	0	0,00
ptg000614l	24667	In assembly	23	0	0,00
ptg000500l	22590	In assembly	12	0	0,00
ptg000025l	10395870	Not in assembly	1	44591	0,70
ptg000051l	8081480	Not in assembly	1	36076	0,73
ptg000100l	6572231	Not in assembly	4	29483	0,74
ptg000049l	5886805	Not in assembly	10	25873	0,72
ptg000073l	5856792	Not in assembly	9	24005	0,68
ptg000011l	5262259	Not in assembly	0	24009	0,75
ptg000054l	5180355	Not in assembly	0	24024	0,76
ptg000067l	5091932	Not in assembly	0	20355	0,66
ptg000040l	4406435	Not in assembly	7	20722	0,77
ptg000007l	3908132	Not in assembly	3	17267	0,73
ptg000068l	3843893	Not in assembly	6	16458	0,70
ptg000130l	3621343	Not in assembly	0	16007	0,73
ptg000074l	3589998	Not in assembly	0	15804	0,72
ptg000087l	3163889	Not in assembly	0	15113	0,79
ptg000089l	3106993	Not in assembly	0	13732	0,73
ptg000066l	2904791	Not in assembly	5	13614	0,77
ptg000014l	2559024	Not in assembly	7	11679	0,75
ptg000064l	2283672	Not in assembly	0	11089	0,80
ptg000108l	2186041	Not in assembly	0	10690	0,80
ptg000092l	1956628	Not in assembly	0	8608	0,72
ptg000113l	1922342	Not in assembly	5	8646	0,74
ptg000193l	1922308	Not in assembly	2	2729	0,23
ptg000151l	1860087	Not in assembly	0	8599	0,76
ptg000034l	1845314	Not in assembly	0	7471	0,66
ptg000050l	1718546	Not in assembly	0	0	0,00
ptg000032l	1690284	Not in assembly	0	7573	0,74
ptg000075l	1676547	Not in assembly	0	7811	0,77
ptg000120l	1566981	Not in assembly	0	7377	0,77
ptg000024l	1430088	Not in assembly	0	6217	0,71
ptg000136l	1424603	Not in assembly	0	6357	0,73
ptg000106l	1313966	Not in assembly	0	5934	0,74

ptg000085l	1297837	Not in assembly	0	6104	0,77
ptg000062l	1288813	Not in assembly	0	6146	0,78
ptg000176l	1281092	Not in assembly	0	5471	0,70
ptg000211l	1264531	Not in assembly	0	5852	0,76
ptg000088l	1256748	Not in assembly	0	5990	0,78
ptg000104l	1238787	Not in assembly	0	5766	0,77
ptg000056l	1184652	Not in assembly	0	5407	0,75
ptg000126l	1156473	Not in assembly	0	5319	0,75
ptg000090l	1110547	Not in assembly	0	4950	0,73
ptg000114l	1084180	Not in assembly	0	4749	0,72
ptg000044l	1079234	Not in assembly	0	4939	0,75
ptg000015l	1074961	Not in assembly	0	4962	0,76
ptg000018l	1068149	Not in assembly	0	4757	0,73
ptg000078l	1036428	Not in assembly	0	4936	0,78
ptg000158l	1006862	Not in assembly	0	4577	0,75
ptg000098l	988208	Not in assembly	0	4693	0,78
ptg000267l	971371	Not in assembly	0	4469	0,76
ptg000213l	887666	Not in assembly	0	4327	0,80
ptg000053l	811358	Not in assembly	0	3485	0,70
ptg000097l	777334	Not in assembly	0	3335	0,70
ptg000059l	775387	Not in assembly	0	3659	0,77
ptg000094l	738787	Not in assembly	0	3468	0,77
ptg000199l	724576	Not in assembly	0	3404	0,77
ptg000063l	716369	Not in assembly	0	3493	0,80
ptg000129l	680079	Not in assembly	0	3117	0,75
ptg000116l	670382	Not in assembly	0	3106	0,76
ptg000121l	660441	Not in assembly	0	3068	0,76
ptg000035l	646817	Not in assembly	0	3019	0,77
ptg000096l	594209	Not in assembly	0	0	0,00
ptg000152l	590308	Not in assembly	0	2792	0,78
ptg000028l	580311	Not in assembly	0	2822	0,80
ptg000093l	568283	Not in assembly	0	2540	0,73
ptg000043l	561382	Not in assembly	0	2721	0,80
ptg000038l	546753	Not in assembly	0	2529	0,76
ptg000186l	537662	Not in assembly	0	0	0,00
ptg000133l	532681	Not in assembly	0	2625	0,81
ptg000076l	527702	Not in assembly	0	2402	0,75
ptg000235l	524959	Not in assembly	0	2441	0,76
ptg000257l	524603	Not in assembly	0	2498	0,78
ptg000182l	523867	Not in assembly	0	2446	0,76
ptg000109l	511458	Not in assembly	0	2387	0,77
ptg000177l	497895	Not in assembly	0	2436	0,81
ptg000127l	497554	Not in assembly	0	2248	0,74
ptg000184l	490759	Not in assembly	0	2340	0,78
ptg000388l	488132	Not in assembly	0	2213	0,74
ptg000128l	485598	Not in assembly	0	2264	0,77
ptg000197l	471589	Not in assembly	0	2417	0,84
ptg000147l	468996	Not in assembly	0	0	0,00
ptg000229l	468900	Not in assembly	0	2298	0,81

ptg000115l	466183	Not in assembly	0	0	0,00
ptg000118l	465240	Not in assembly	0	2116	0,75
ptg000259l	461691	Not in assembly	0	2204	0,78
ptg000070l	459566	Not in assembly	0	2240	0,80
ptg000153l	454565	Not in assembly	0	2039	0,74
ptg000155l	452951	Not in assembly	0	2127	0,77
ptg000037l	446734	Not in assembly	0	1960	0,72
ptg000170l	433386	Not in assembly	0	1941	0,74
ptg000327l	432635	Not in assembly	0	2252	0,86
ptg000124l	422641	Not in assembly	0	2040	0,79
ptg000001l	419306	Not in assembly	0	2068	0,81
ptg000165l	405545	Not in assembly	0	1787	0,72
ptg000026l	403608	Not in assembly	0	1954	0,79
ptg000167l	392659	Not in assembly	0	1896	0,79
ptg000304l	391381	Not in assembly	0	1860	0,78
ptg000214l	384589	Not in assembly	0	1733	0,74
ptg000175l	372308	Not in assembly	0	1808	0,80
ptg000080l	368092	Not in assembly	0	1784	0,79
ptg000222l	361668	Not in assembly	0	1621	0,73
ptg000248l	360381	Not in assembly	0	1742	0,79
ptg000318l	357318	Not in assembly	0	1710	0,79
ptg000072l	356993	Not in assembly	0	1683	0,77
ptg000164l	356501	Not in assembly	0	1752	0,81
ptg000047l	351567	Not in assembly	0	1620	0,76
ptg000042l	345137	Not in assembly	0	202	0,09
ptg000296l	343458	Not in assembly	0	1493	0,71
ptg000185l	339152	Not in assembly	0	1532	0,74
ptg000203l	328842	Not in assembly	0	1307	0,65
ptg000281l	326709	Not in assembly	4	29	0,02
ptg000110l	322964	Not in assembly	0	0	0,00
ptg000356l	322616	Not in assembly	0	1560	0,79
ptg000145l	320972	Not in assembly	0	1580	0,81
ptg000183l	314762	Not in assembly	0	1557	0,81
ptg000250l	307075	Not in assembly	0	1422	0,76
ptg000275l	305176	Not in assembly	0	1461	0,79
ptg000218l	297780	Not in assembly	0	44	0,04
ptg000366l	293149	Not in assembly	0	1433	0,80
ptg000427l	290808	Not in assembly	0	56	0,05
ptg000349l	290317	Not in assembly	0	1360	0,77
ptg000270l	285906	Not in assembly	0	1328	0,76
ptg000160l	279617	Not in assembly	0	1340	0,79
ptg000343l	276429	Not in assembly	0	0	0,00
ptg000220l	275893	Not in assembly	2	13	0,01
ptg000163l	271400	Not in assembly	0	1243	0,75
ptg000188l	267609	Not in assembly	0	169	0,09
ptg000216l	262024	Not in assembly	0	7	0,00
ptg000362l	260182	Not in assembly	0	1335	0,84
ptg000017l	253722	Not in assembly	0	1117	0,72
ptg000134l	252353	Not in assembly	0	1184	0,77

ptg000283l	250011	Not in assembly	0	1218	0,80
ptg000174l	245862	Not in assembly	0	1156	0,77
ptg000233l	243028	Not in assembly	0	1198	0,81
ptg000065l	242163	Not in assembly	0	0	0,00
ptg000142l	241074	Not in assembly	0	1084	0,74
ptg000132l	237971	Not in assembly	0	1107	0,76
ptg000192c	232129	Not in assembly	0	1094	0,77
ptg000481l	228167	Not in assembly	0	1134	0,81
ptg000323l	227978	Not in assembly	0	40	0,05
ptg000272l	227402	Not in assembly	0	0	0,00
ptg000340l	225249	Not in assembly	0	1103	0,80
ptg000159l	224176	Not in assembly	0	1018	0,74
ptg000205l	223432	Not in assembly	0	0	0,00
ptg000300l	221232	Not in assembly	0	991	0,74
ptg000137l	221142	Not in assembly	0	0	0,00
ptg000241l	220608	Not in assembly	0	0	0,00
ptg000122l	219331	Not in assembly	0	1183	0,89
ptg000143l	218579	Not in assembly	0	964	0,73
ptg000161l	217341	Not in assembly	0	0	0,00
ptg000298l	213190	Not in assembly	0	0	0,00
ptg000154l	213057	Not in assembly	0	908	0,70
ptg000434l	212017	Not in assembly	0	1038	0,81
ptg000169l	211889	Not in assembly	0	0	0,00
ptg000289l	211858	Not in assembly	0	1064	0,82
ptg000135l	211663	Not in assembly	0	877	0,68
ptg000360l	208658	Not in assembly	0	995	0,78
ptg000171l	208381	Not in assembly	0	0	0,00
ptg000013l	207939	Not in assembly	0	914	0,72
ptg000254l	207419	Not in assembly	0	1021	0,81
ptg000240l	205407	Not in assembly	0	938	0,75
ptg000310l	205373	Not in assembly	0	967	0,77
ptg000234l	204192	Not in assembly	0	979	0,79
ptg000269l	198998	Not in assembly	0	987	0,81
ptg000256l	191947	Not in assembly	0	0	0,00
ptg000402l	191944	Not in assembly	0	883	0,76
ptg000119l	191793	Not in assembly	0	874	0,75
ptg000131l	191266	Not in assembly	0	558	0,48
ptg000333l	190778	Not in assembly	0	934	0,81
ptg000313l	190689	Not in assembly	0	872	0,75
ptg000459l	189259	Not in assembly	7	847	0,74
ptg000206l	185278	Not in assembly	0	768	0,68
ptg000189l	184537	Not in assembly	0	857	0,76
ptg000263l	183806	Not in assembly	0	916	0,82
ptg000201l	182304	Not in assembly	0	837	0,75
ptg000146l	180670	Not in assembly	0	58	0,08
ptg000271l	178886	Not in assembly	0	923	0,85
ptg000178l	178183	Not in assembly	0	860	0,79
ptg000348l	177789	Not in assembly	0	898	0,83
ptg000309l	175500	Not in assembly	0	0	0,00

ptg000058l	173077	Not in assembly	0	823	0,78
ptg000355l	172555	Not in assembly	0	756	0,72
ptg000149l	172174	Not in assembly	0	788	0,75
ptg000210l	170413	Not in assembly	0	781	0,76
ptg000239l	168659	Not in assembly	0	753	0,73
ptg000496l	161905	Not in assembly	9	4	0,00
ptg000279l	160641	Not in assembly	0	0	0,00
ptg000335l	160505	Not in assembly	0	750	0,77
ptg000344l	160111	Not in assembly	0	748	0,77
ptg000141l	158326	Not in assembly	0	0	0,00
ptg000358c	157558	Not in assembly	0	695	0,72
ptg000421l	157114	Not in assembly	0	734	0,77
ptg000079l	156019	Not in assembly	0	731	0,77
ptg000338l	155146	Not in assembly	0	754	0,80
ptg000411l	152887	Not in assembly	0	699	0,75
ptg000223l	150609	Not in assembly	0	706	0,77
ptg000354l	150091	Not in assembly	0	551	0,60
ptg000462l	149369	Not in assembly	0	720	0,79
ptg000168l	149014	Not in assembly	0	724	0,80
ptg000373l	147913	Not in assembly	0	680	0,75
ptg000238l	146699	Not in assembly	0	0	0,00
ptg000260l	142725	Not in assembly	0	0	0,00
ptg000425l	141017	Not in assembly	0	712	0,83
ptg000398l	140870	Not in assembly	0	598	0,70
ptg000242l	138364	Not in assembly	0	0	0,00
ptg000383l	138117	Not in assembly	0	633	0,75
ptg000443l	136355	Not in assembly	0	661	0,80
ptg000274l	135896	Not in assembly	0	0	0,00
ptg000312l	135004	Not in assembly	9	0	0,00
ptg000027l	132822	Not in assembly	0	0	0,00
ptg000157l	128812	Not in assembly	0	641	0,82
ptg000286l	127089	Not in assembly	0	651	0,84
ptg000376l	125399	Not in assembly	0	588	0,77
ptg000217l	124064	Not in assembly	0	549	0,73
ptg000522l	122862	Not in assembly	0	668	0,90
ptg000487l	121955	Not in assembly	0	490	0,66
ptg000448l	121877	Not in assembly	0	538	0,73
ptg000401c	121621	Not in assembly	0	0	0,00
ptg000276l	119532	Not in assembly	0	575	0,79
ptg000485l	118585	Not in assembly	0	0	0,00
ptg000357l	117353	Not in assembly	0	583	0,82
ptg000467l	116613	Not in assembly	0	0	0,00
ptg000442l	116125	Not in assembly	0	573	0,81
ptg000295l	110045	Not in assembly	0	0	0,00
ptg000394l	109933	Not in assembly	0	0	0,00
ptg000207l	108246	Not in assembly	0	461	0,70
ptg000247l	107652	Not in assembly	0	0	0,00
ptg000314l	106891	Not in assembly	0	524	0,81
ptg000453l	106698	Not in assembly	0	530	0,82

ptg000320l	106372	Not in assembly	0	486	0,75
ptg000345l	105208	Not in assembly	0	475	0,74
ptg000209l	103893	Not in assembly	0	493	0,78
ptg000445l	103753	Not in assembly	0	395	0,62
ptg000347l	101855	Not in assembly	0	464	0,75
ptg000375l	101059	Not in assembly	0	497	0,81
ptg000077l	99753	Not in assembly	0	513	0,85
ptg000191l	98794	Not in assembly	0	0	0,00
ptg000474l	98337	Not in assembly	0	456	0,76
ptg000307l	98328	Not in assembly	0	438	0,73
ptg000023c	98234	Not in assembly	0	472	0,79
ptg000524l	97609	Not in assembly	0	437	0,74
ptg000395l	97396	Not in assembly	0	436	0,74
ptg000439l	97173	Not in assembly	0	475	0,80
ptg000454l	97135	Not in assembly	0	474	0,80
ptg000482l	96229	Not in assembly	0	454	0,77
ptg000390l	95794	Not in assembly	0	0	0,00
ptg000324l	95746	Not in assembly	0	408	0,70
ptg000452l	95568	Not in assembly	0	473	0,81
ptg000190l	95152	Not in assembly	0	432	0,75
ptg000230l	93748	Not in assembly	0	421	0,74
ptg000423l	93733	Not in assembly	0	490	0,86
ptg000339l	93168	Not in assembly	0	0	0,00
ptg000156l	92763	Not in assembly	0	472	0,84
ptg000224l	90971	Not in assembly	5	0	0,00
ptg000123l	90614	Not in assembly	0	462	0,84
ptg000521l	90603	Not in assembly	0	0	0,00
ptg000393l	90484	Not in assembly	0	485	0,88
ptg000495l	86894	Not in assembly	6	0	0,00
ptg000221l	86451	Not in assembly	0	0	0,00
ptg000438l	86296	Not in assembly	0	405	0,77
ptg000470l	85322	Not in assembly	0	394	0,76
ptg000498l	85171	Not in assembly	0	0	0,00
ptg000308c	84936	Not in assembly	3	0	0,00
ptg000086l	84061	Not in assembly	9	0	0,00
ptg000363l	83893	Not in assembly	0	0	0,00
ptg000369l	83782	Not in assembly	0	2	0,00
ptg000195l	82798	Not in assembly	0	0	0,00
ptg000291l	82682	Not in assembly	0	383	0,76
ptg000258l	82229	Not in assembly	0	366	0,73
ptg000292l	81820	Not in assembly	0	440	0,88
ptg000172l	80791	Not in assembly	0	0	0,00
ptg000367l	79041	Not in assembly	0	0	0,00
ptg000315l	78865	Not in assembly	0	372	0,78
ptg000033l	78764	Not in assembly	0	391	0,81
ptg000208l	78538	Not in assembly	0	131	0,28
ptg000084l	78477	Not in assembly	0	389	0,81
ptg000365l	78281	Not in assembly	0	380	0,80
ptg000336l	77612	Not in assembly	0	15	0,05

ptg000432l	77484	Not in assembly	0	0	0,00
ptg000288l	77442	Not in assembly	0	376	0,80
ptg000410l	77175	Not in assembly	0	369	0,79
ptg000403l	76893	Not in assembly	0	390	0,84
ptg000457l	75778	Not in assembly	0	356	0,77
ptg000029c	75186	Not in assembly	0	319	0,69
ptg000436l	74685	Not in assembly	0	434	0,96
ptg000541l	74412	Not in assembly	0	372	0,82
ptg000577l	74221	Not in assembly	0	0	0,00
ptg000261l	73761	Not in assembly	0	345	0,77
ptg000290l	73433	Not in assembly	0	401	0,90
ptg000280l	73038	Not in assembly	0	0	0,00
ptg000542l	72534	Not in assembly	0	0	0,00
ptg000514l	72172	Not in assembly	0	0	0,00
ptg000138l	71282	Not in assembly	0	356	0,82
ptg000553l	69629	Not in assembly	0	363	0,86
ptg000301l	68243	Not in assembly	0	14	0,03
ptg000284l	66757	Not in assembly	4	0	0,00
ptg000460l	66293	Not in assembly	0	356	0,89
ptg000506l	65555	Not in assembly	0	288	0,72
ptg000449l	65100	Not in assembly	0	0	0,00
ptg000469l	64918	Not in assembly	0	0	0,00
ptg000255l	64144	Not in assembly	0	317	0,81
ptg000287l	63955	Not in assembly	0	299	0,77
ptg000554l	63891	Not in assembly	0	0	0,00
ptg000277l	62918	Not in assembly	0	0	0,00
ptg000400l	61929	Not in assembly	0	0	0,00
ptg000501l	61754	Not in assembly	0	296	0,79
ptg000262l	61128	Not in assembly	0	0	0,00
ptg000430l	60471	Not in assembly	0	295	0,80
ptg000510l	59750	Not in assembly	0	13	0,04
ptg000520l	58202	Not in assembly	0	278	0,78
ptg000409l	58034	Not in assembly	0	276	0,78
ptg000294l	56599	Not in assembly	0	295	0,86
ptg000507l	56120	Not in assembly	0	0	0,00
ptg000471l	55724	Not in assembly	0	279	0,82
ptg000265l	55645	Not in assembly	0	257	0,76
ptg000227l	54868	Not in assembly	0	294	0,88
ptg000412l	54803	Not in assembly	0	0	0,00
ptg000297l	54741	Not in assembly	0	239	0,72
ptg000252l	54463	Not in assembly	0	0	0,00
ptg000446l	54395	Not in assembly	0	271	0,82
ptg000332c	54370	Not in assembly	0	238	0,72
ptg000575l	54103	Not in assembly	0	0	0,00
ptg000381l	53698	Not in assembly	0	240	0,73
ptg000226l	53514	Not in assembly	0	250	0,77
ptg000350l	53500	Not in assembly	0	0	0,00
ptg000479l	53285	Not in assembly	0	0	0,00
ptg000574l	51856	Not in assembly	0	0	0,00

ptg000244l	51653	Not in assembly	1	0	0,00
ptg000490l	51583	Not in assembly	0	0	0,00
ptg000502l	51554	Not in assembly	0	257	0,82
ptg000447l	51546	Not in assembly	0	0	0,00
ptg000547l	51515	Not in assembly	0	257	0,82
ptg000125l	51104	Not in assembly	0	224	0,72
ptg000480l	51084	Not in assembly	0	254	0,82
ptg000465l	51067	Not in assembly	0	249	0,80
ptg000237l	50582	Not in assembly	0	2	0,00
ptg000584l	50320	Not in assembly	0	230	0,75
ptg000455l	49964	Not in assembly	0	215	0,71
ptg000407l	49942	Not in assembly	0	230	0,75
ptg000456l	49654	Not in assembly	0	0	0,00
ptg000537l	49606	Not in assembly	0	218	0,72
ptg000517l	49315	Not in assembly	0	0	0,00
ptg000316c	49296	Not in assembly	0	239	0,79
ptg000546l	48915	Not in assembly	0	248	0,83
ptg000334l	48798	Not in assembly	0	2	0,00
ptg000387l	48511	Not in assembly	0	0	0,00
ptg000180l	48142	Not in assembly	0	0	0,00
ptg000540l	47457	Not in assembly	0	0	0,00
ptg000346l	46837	Not in assembly	0	0	0,00
ptg000370l	46824	Not in assembly	0	0	0,00
ptg000382l	46792	Not in assembly	0	0	0,00
ptg000468l	46499	Not in assembly	0	0	0,00
ptg000561l	46001	Not in assembly	0	225	0,80
ptg000322l	45640	Not in assembly	0	213	0,77
ptg000557l	45610	Not in assembly	0	0	0,00
ptg000441l	45202	Not in assembly	0	0	0,00
ptg000477l	45105	Not in assembly	0	0	0,00
ptg000509l	45099	Not in assembly	0	221	0,80
ptg000573l	44879	Not in assembly	0	0	0,00
ptg000617l	44855	Not in assembly	0	0	0,00
ptg000535l	44853	Not in assembly	0	0	0,00
ptg000505l	44691	Not in assembly	0	212	0,78
ptg000572l	44208	Not in assembly	0	0	0,00
ptg000473l	43957	Not in assembly	0	87	0,18
ptg000475l	43104	Not in assembly	0	218	0,83
ptg000598l	43083	Not in assembly	0	0	0,00
ptg000549l	42854	Not in assembly	0	0	0,00
ptg000466l	42712	Not in assembly	0	228	0,87
ptg000580l	42683	Not in assembly	0	0	0,00
ptg000497l	42542	Not in assembly	0	0	0,00
ptg000523l	42442	Not in assembly	0	0	0,00
ptg000337l	42084	Not in assembly	0	223	0,87
ptg000560l	41711	Not in assembly	0	0	0,00
ptg000579l	41161	Not in assembly	0	0	0,00
ptg000278l	41142	Not in assembly	0	0	0,00
ptg000567l	41115	Not in assembly	5	0	0,00

ptg000534l	40278	Not in assembly	0	0	0,00
ptg000504l	40157	Not in assembly	0	222	0,91
ptg000305l	40016	Not in assembly	0	0	0,00
ptg000499l	39616	Not in assembly	0	115	0,48
ptg000636l	39472	Not in assembly	0	194	0,81
ptg000491l	39002	Not in assembly	0	152	0,64
ptg000351l	38737	Not in assembly	0	0	0,00
ptg000645l	38673	Not in assembly	0	174	0,74
ptg000625l	38610	Not in assembly	0	0	0,00
ptg000559l	38301	Not in assembly	0	209	0,90
ptg000091l	38279	Not in assembly	0	0	0,00
ptg000605l	38122	Not in assembly	0	0	0,00
ptg000268l	37776	Not in assembly	0	0	0,00
ptg000608l	37727	Not in assembly	0	0	0,00
ptg000528l	37426	Not in assembly	0	17	0,07
ptg000476l	37388	Not in assembly	0	194	0,86
ptg000231l	37275	Not in assembly	0	149	0,66
ptg000655l	36968	Not in assembly	0	191	0,85
ptg000431l	36943	Not in assembly	0	156	0,69
ptg000385l	36876	Not in assembly	0	155	0,69
ptg000519l	36826	Not in assembly	0	0	0,00
ptg000415l	36291	Not in assembly	0	14	0,06
ptg000451l	36163	Not in assembly	0	0	0,00
ptg000458l	36153	Not in assembly	8	0	0,00
ptg000582l	36028	Not in assembly	0	0	0,00
ptg000428l	35983	Not in assembly	0	202	0,92
ptg000647l	35697	Not in assembly	0	157	0,72
ptg000440l	35684	Not in assembly	0	0	0,00
ptg000653l	34479	Not in assembly	0	174	0,83
ptg000187l	34389	Not in assembly	0	0	0,00
ptg000527l	34202	Not in assembly	0	155	0,74
ptg000600l	34114	Not in assembly	0	0	0,00
ptg000526l	34096	Not in assembly	0	0	0,00
ptg000613l	34054	Not in assembly	0	0	0,00
ptg000610l	33963	Not in assembly	0	8	0,04
ptg000585l	33875	Not in assembly	0	0	0,00
ptg000299l	33810	Not in assembly	0	0	0,00
ptg000377l	33046	Not in assembly	0	0	0,00
ptg000488l	32894	Not in assembly	0	168	0,84
ptg000634l	32427	Not in assembly	0	173	0,88
ptg000629l	32380	Not in assembly	0	0	0,00
ptg000592l	32277	Not in assembly	0	0	0,00
ptg000273l	32116	Not in assembly	0	0	0,00
ptg000631l	31998	Not in assembly	0	180	0,93
ptg000662l	31977	Not in assembly	0	161	0,83
ptg000539l	31950	Not in assembly	0	0	0,00
ptg000609l	31908	Not in assembly	0	0	0,00
ptg000364l	31907	Not in assembly	0	156	0,80
ptg000544l	31900	Not in assembly	0	156	0,81

ptg000538l	31799	Not in assembly	0	0	0,00
ptg000472l	31581	Not in assembly	0	0	0,00
ptg000391l	31530	Not in assembly	0	259	0,90
ptg000511l	31440	Not in assembly	0	17	0,06
ptg000464l	31437	Not in assembly	0	0	0,01
ptg000493l	30965	Not in assembly	0	151	0,80
ptg000552l	30817	Not in assembly	0	0	0,00
ptg000374l	30743	Not in assembly	0	0	0,00
ptg000418l	30673	Not in assembly	0	272	0,97
ptg000590l	30601	Not in assembly	0	0	0,00
ptg000204l	30515	Not in assembly	0	0	0,00
ptg000543l	30463	Not in assembly	0	0	0,00
ptg000379l	30443	Not in assembly	0	142	0,76
ptg000181l	30254	Not in assembly	0	0	0,00
ptg000325l	30253	Not in assembly	0	0	0,00
ptg000532l	30210	Not in assembly	0	0	0,00
ptg000569l	30191	Not in assembly	0	180	0,98
ptg000489l	30169	Not in assembly	0	0	0,00
ptg000551l	30018	Not in assembly	0	118	0,64
ptg000422l	29963	Not in assembly	0	139	0,76
ptg000565l	29881	Not in assembly	0	0	0,00
ptg000583l	29819	Not in assembly	1	0	0,00
ptg000414l	29793	Not in assembly	0	144	0,80
ptg000417l	29564	Not in assembly	0	0	0,00
ptg000494l	29286	Not in assembly	0	111	0,62
ptg000550l	29141	Not in assembly	0	124	0,70
ptg000587l	29114	Not in assembly	0	0	0,00
ptg000371l	29067	Not in assembly	0	0	0,00
ptg000484l	28479	Not in assembly	0	0	0,00
ptg000642l	28325	Not in assembly	0	109	0,63
ptg000659l	28296	Not in assembly	0	141	0,82
ptg000558l	28220	Not in assembly	0	140	0,82
ptg000562l	28209	Not in assembly	0	137	0,80
ptg000435l	27827	Not in assembly	0	0	0,00
ptg000616l	27804	Not in assembly	0	136	0,80
ptg000619l	27738	Not in assembly	0	0	0,00
ptg000632l	27594	Not in assembly	0	120	0,72
ptg000628l	27427	Not in assembly	0	124	0,74
ptg000618l	27329	Not in assembly	5	0	0,00
ptg000512l	27253	Not in assembly	0	0	0,00
ptg000566l	27036	Not in assembly	0	0	0,00
ptg000594l	27021	Not in assembly	0	2	0,01
ptg000623l	27004	Not in assembly	0	114	0,69
ptg000219l	26982	Not in assembly	0	0	0,00
ptg000660l	26732	Not in assembly	0	128	0,79
ptg000530l	26567	Not in assembly	0	158	0,98
ptg000386l	26555	Not in assembly	0	0	0,00
ptg000486l	26421	Not in assembly	0	0	0,00
ptg000361l	26404	Not in assembly	0	0	0,00

ptg000601l	26382	Not in assembly	0	0	0,00
ptg000555l	26367	Not in assembly	0	0	0,00
ptg000586l	26251	Not in assembly	0	0	0,00
ptg000578l	25909	Not in assembly	7	0	0,00
ptg000646l	25811	Not in assembly	0	0	0,00
ptg000666l	25645	Not in assembly	0	125	0,80
ptg000513c	25638	Not in assembly	0	123	0,78
ptg000508c	25472	Not in assembly	0	11	0,11
ptg000651l	25447	Not in assembly	0	134	0,87
ptg000603l	25426	Not in assembly	0	0	0,00
ptg000656l	25413	Not in assembly	0	121	0,78
ptg000529l	25314	Not in assembly	0	0	0,00
ptg000536l	25262	Not in assembly	0	0	0,00
ptg000589l	25225	Not in assembly	0	0	0,00
ptg000581l	25190	Not in assembly	0	0	0,00
ptg000612l	25172	Not in assembly	0	0	0,00
ptg000380l	25146	Not in assembly	0	213	0,93
ptg000643l	25104	Not in assembly	0	121	0,79
ptg000515l	25099	Not in assembly	1	0	0,00
ptg000622l	25019	Not in assembly	0	11	0,11
ptg000670l	24950	Not in assembly	0	129	0,85
ptg000317l	24949	Not in assembly	0	120	0,79
ptg000602l	24796	Not in assembly	0	121	0,80
ptg000649l	24746	Not in assembly	0	0	0,00
ptg000285l	24627	Not in assembly	0	121	0,81
ptg000593l	24600	Not in assembly	0	198	0,87
ptg000516l	24530	Not in assembly	0	0	0,00
ptg000433l	24205	Not in assembly	0	122	0,83
ptg000212l	24143	Not in assembly	0	0	0,00
ptg000675l	24031	Not in assembly	0	114	0,77
ptg000563l	24029	Not in assembly	0	0	0,00
ptg000637l	23963	Not in assembly	0	125	0,86
ptg000633l	23869	Not in assembly	0	0	0,00
ptg000548l	23863	Not in assembly	0	118	0,81
ptg000639l	23729	Not in assembly	0	10	0,11
ptg000352l	23670	Not in assembly	0	0	0,00
ptg000416l	23665	Not in assembly	5	49	0,34
ptg000568l	23664	Not in assembly	0	0	0,00
ptg000596l	23650	Not in assembly	0	116	0,81
ptg000483l	23601	Not in assembly	0	0	0,00
ptg000678l	23361	Not in assembly	0	0	0,00
ptg000638l	23346	Not in assembly	0	138	0,98
ptg000139l	23312	Not in assembly	1	0	0,00
ptg000626l	23215	Not in assembly	0	125	0,88
ptg000408l	22976	Not in assembly	0	0	0,00
ptg000611l	22835	Not in assembly	0	0	0,00
ptg000478l	22673	Not in assembly	0	2	0,01
ptg000564l	22638	Not in assembly	0	0	0,00
ptg000635l	22441	Not in assembly	0	132	0,97

ptg000595l	22388	Not in assembly	0	108	0,80
ptg000571l	22376	Not in assembly	0	0	0,00
ptg000492l	22206	Not in assembly	0	0	0,00
ptg000641l	22171	Not in assembly	0	0	0,00
ptg000545l	21984	Not in assembly	0	0	0,00
ptg000661l	21935	Not in assembly	0	0	0,00
ptg000640l	21829	Not in assembly	0	130	0,98
ptg000450l	21594	Not in assembly	0	94	0,72
ptg000392l	21394	Not in assembly	0	95	0,73
ptg000677l	21250	Not in assembly	0	115	0,89
ptg000615l	21188	Not in assembly	0	0	0,00
ptg000597l	21120	Not in assembly	0	0	0,00
ptg000679l	21110	Not in assembly	0	126	0,98
ptg000665l	21091	Not in assembly	0	0	0,00
ptg000652l	21023	Not in assembly	0	0	0,00
ptg000518l	20930	Not in assembly	0	0	0,00
ptg000657l	20744	Not in assembly	0	0	0,00
ptg000667l	20713	Not in assembly	0	105	0,84
ptg000676l	20675	Not in assembly	0	95	0,75
ptg000624l	20408	Not in assembly	0	113	0,91
ptg000669l	20359	Not in assembly	0	0	0,00
ptg000630l	20262	Not in assembly	0	121	0,99
ptg000658l	20165	Not in assembly	0	104	0,85
ptg000673l	20038	Not in assembly	0	0	0,00
ptg000654l	19944	Not in assembly	0	0	0,00
ptg000331l	19751	Not in assembly	0	116	0,64
ptg000668l	19683	Not in assembly	0	116	0,97
ptg000674l	19320	Not in assembly	0	80	0,68
ptg000648l	19168	Not in assembly	0	115	0,99
ptg000664l	19040	Not in assembly	0	103	0,89
ptg000650l	18771	Not in assembly	0	81	0,71
ptg000663l	18708	Not in assembly	0	106	0,93
ptg000591l	18499	Not in assembly	0	0	0,00
ptg000405l	18467	Not in assembly	0	0	0,00
ptg000533l	18170	Not in assembly	0	0	0,00
ptg000588l	18159	Not in assembly	0	0	0,00
ptg000576l	18107	Not in assembly	0	0	0,00
ptg000404l	17950	Not in assembly	0	68	0,62
ptg000672l	17714	Not in assembly	0	85	0,79
ptg000620l	17658	Not in assembly	0	0	0,01
ptg000606l	16957	Not in assembly	0	0	0,00
ptg000607l	16841	Not in assembly	0	0	0,00
ptg000627l	16775	Not in assembly	0	82	0,80
ptg000671l	16368	Not in assembly	0	95	0,96
ptg000644l	14979	Not in assembly	0	0	0,00
ptg000604l	14717	Not in assembly	0	0	0,00
ptg000621l	14065	Not in assembly	0	0	0,00
ptg000599l	13227	Not in assembly	0	2	0,02
