# Supplementary material for: Reference-Guided De Novo Genome Assembly of the Flour Beetle Tribolium freemani
Source: Int J Mol Sci. 2022 May 24;23(11):5869. doi: 10.3390/ijms23115869 (PMC9180572; doi:10.3390/ijms23115869)
Supplement: Supplementary file 1 [file ijms-23-05869-s001.zip › Table S2.pdf]

Table S2. Statistics of contig orientation into chromosome level scaffolds using RagTag algorithm.

	**No. of contigs**	**Total length (bp)**
**Placed sequences**	99	261,849,599
**Unplaced sequences**	11	7,168,944
**Total**	110	269,018,543
