# Supplementary material for: Reference-Guided De Novo Genome Assembly of the Flour Beetle Tribolium freemani
Source: Int J Mol Sci. 2022 May 24;23(11):5869. doi: 10.3390/ijms23115869 (PMC9180572; doi:10.3390/ijms23115869)
Supplement: Supplementary file 1 [file ijms-23-05869-s001.zip › Table S3.pdf]

Table S3. Orientation scores obtained by RagTag algorithm used for optimal positioning of 99 contigs that were placed into ten *T. freemani* linkage groups.

Query	Grouping confidence	Location confidence	Orientation confidence
ptg000095l	0,282362872	0,003131799	0,661787445
ptg000396l	1	1	1
ptg000061l	0,748019496	0,345728428	0,449208572
ptg000312l	0,585727069	1	1
ptg000069l	0,723874362	0,55532304	0,243468999
ptg000102l	0,414721037	0,201463235	0,736722249
ptg000082l	0,712977558	0,373185265	0,829946981
ptg000010l	0,722713119	0,397163278	0,820206699
ptg000531l	1	1	1
ptg000148l	0,998838354	0,951774536	0,995757831
ptg000368l	1	0,9570073	0,98293186
ptg000330l	0,986167369	1	1
ptg000419l	0,99860062	0,943270051	0,972889024
ptg000461l	1	0,982042959	1
ptg000503l	1	0,985786958	0,989826461
ptg000117l	0,977406168	0,185363949	0,999170153
ptg000329l	1	0,008132964	1
ptg000003l	0,984879426	0,940130016	0,755115327
ptg000444l	0,998919819	0,978162186	0,977257879
ptg000567l	1	1	1
ptg000081l	0,956242944	0,336864242	0,987869528
ptg000112l	0,729717703	0,940047908	0,995152362
ptg000282l	0,734238289	0,001982547	0,513485681
ptg000021l	0,916067206	0,115443094	0,160772172
ptg000326l	1	1	1
ptg000420l	1	1	1
ptg000525l	1	1	1
ptg000041l	0,922855357	0,288146229	0,818979465
ptg000019l	0,969990345	0,148107559	0,808289872
ptg000426l	1	1	1
ptg000264l	0,929385448	0,012157895	0,94121666
ptg000284l	0,935817394	1	1
ptg000458l	1	1	1
ptg000406l	1	1	1
ptg000046l	0,935163498	0,038421865	0,965670813
ptg000321l	0,998962875	1	1
ptg000384l	0,606120222	0,07976616	1
ptg000228l	0,985282263	0,013775343	0,8943217
ptg000006l	0,951934424	0,188504737	0,315147034
ptg000045l	0,338299399	0,00023801	0,517046968
ptg000083l	0,982631229	0,149056691	0,921074341
ptg000251l	0,982860139	0,051680649	0,900402357
ptg000039l	0,859728185	0,127162311	0,959260302
ptg000144l	0,945222044	0,033862795	0,382240711
ptg000150l	0,79303814	0,025433549	0,715867987
ptg000105l	0,897879341	0,351663524	0,441262073
ptg000437l	0,775430104	0,008586812	0,617681795
ptg000232l	0,977236585	0,294405636	0,756493708

ptg000570l	0,961231367	1	1
ptg000225l	1	0,988107995	0,992456959
ptg000057l	1	1	1
ptg000012l	0,951832745	0,915297376	0,874675916
ptg000500l	1	1	1
ptg000306l	0,99955176	0,979082701	1
ptg000162l	0,995357952	0,976040904	1
ptg000341l	0,999433238	0,915409621	0,96480777
ptg000302l	1	1	1
ptg000293l	1	1	1
ptg000328l	1	1	1
ptg000397l	0,999681823	1	1
ptg000016l	0,91065262	0,87368693	0,956626509
ptg000111l	0,977765752	0,987779906	1
ptg000429l	1	1	1
ptg000413l	1	1	1
ptg000266l	1	1	1
ptg000215l	0,993707057	0,560686821	0,807959941
ptg000036l	0,911162587	0,205219886	0,856608446
ptg000020l	0,530657909	0,064258341	0,805332178
ptg000048l	0,824121171	0,594120022	0,559635672
ptg000103l	0,374219575	0,008528544	0,904668811
ptg000496l	0,876184803	0,785480932	1
ptg000245l	0,72212775	0,233015536	0,926815807
ptg000319l	0,94672919	0,191919025	0,915585637
ptg000353l	1	0,922991185	1
ptg000173l	0,999581681	0,99956081	0,999155809
ptg000060l	0,942658347	0,421208425	0,715848464
ptg000071l	0,925205246	0,355543082	0,14976901
ptg000107l	0,349987922	0,012723359	0,947852721
ptg000194l	0,537048595	0,134455885	0,917195544
ptg000030l	0,532948461	1	1
ptg000052l	0,329336245	0,00743164	0,573421654
ptg000073l	0,81767427	0,152326048	0,615989833
ptg000022l	0,879849841	0,133114747	0,789978285
ptg000614l	0,985196052	1	1
ptg000311l	0,453172966	0,019302102	0,950826715
ptg000303l	0,612073553	0,016294219	0,980494912
ptg000463l	1	0,932071975	0,941969237
ptg000008l	0,965965491	0,662803894	0,610037257
ptg000359l	0,511287289	0,955300603	0,986417635
ptg000399l	0,984634528	0,033305431	0,870252777
ptg000253l	0,947074838	0,467621773	0,8115905
ptg000200l	0,993168195	0,023736582	0,499682348
ptg000166l	0,645336404	0,094120637	0,696647924
ptg000556l	1	1	1
ptg000004l	0,828767724	0,152176001	0,555214214
ptg000002l	0,969771287	0,656696728	0,663338592
ptg000031l	0,831225966	0,306078389	0,629765486
ptg000005l	0,973226347	0,546662368	0,42995444
ptg000202l	0,930355862	1	1
