# Supplementary material for: Reference-Guided De Novo Genome Assembly of the Flour Beetle Tribolium freemani
Source: Int J Mol Sci. 2022 May 24;23(11):5869. doi: 10.3390/ijms23115869 (PMC9180572; doi:10.3390/ijms23115869)
Supplement: Supplementary file 1 [file ijms-23-05869-s001.zip › Table S4.pdf]

Table S4. Chromosome sizes in the *T. freemani* Tfree1.0 assembly (*f*LGs) compared to the chromosome sizes in the *T. castaneum* Tcas5.2 assembly (LGs). Chromosome sizes are calculated without ambiguous bases (N) and expressed in Mb.

T. freemani **Tfree1.0 chromosomes**	***f*LG size (Mb)**	***T. castaneum*** **Tcas5.2 chromosomes**	**LG size (Mb)**	***f*LG vs. LG size difference (Mb)**
***f*LG2**	32.31	**LG2**	14.56	17.75
***f*LG3**	42.26	**LG3**	29.60	12.66
***f*LG4**	16.96	**LG4**	11.63	5.33
***f*LG5**	24.98	**LG5**	14.34	10.64
***f*LG6**	23.21	**LG6**	7.98	15.23
***f*LG7**	38.63	**LG7**	15.19	23.44
***f*LG8**	23.81	**LG8**	13.62	10.19
***f*LG9**	32.41	**LG9**	15.01	17.41
***f*LG10**	15.39	**LG10**	6.78	8.61
***f*LGX**	12.03	**LGX**	7.43	4.60
**Total**	262.00	**Total**	136.14	125.86
