# Supplementary material for: Reference-Guided De Novo Genome Assembly of the Flour Beetle Tribolium freemani
Source: Int J Mol Sci. 2022 May 24;23(11):5869. doi: 10.3390/ijms23115869 (PMC9180572; doi:10.3390/ijms23115869)
Supplement: Supplementary file 1 [file ijms-23-05869-s001.zip › Table S5.pdf]

Table S5. Distribution of repeat elements in *T. freemani* Tfree1.0 and *T. castaneum* Tcas5.2 genome assemblies. Linkage groups and unplaced contigs are taken into account for both species.

	Tfree1.0			Tcas5.2		
**Repeat family**	Number of elements	Length (bp)	Genome proportion (%)	Number of elements	Length (bp)	Genome proportion (%)
DNA	57,279	14,051,034	5.22	33,970	8,279,569	4.99
LINE	8,897	2,926,173	1.09	4,684	1,572,720	0.95
LTR	5,563	1,827,084	0.68	2,593	766,028	0.46
Low_complexity	67,068	2,834,397	1.05	10,168	487,704	0.29
Rolling circle	3,692	1,345,124	0.50	1,997	258,101	0.16
SINE	839	149,600	0.06	190	24,455	0.01
Simple_repeat	138,612	6,537,487	2.43	36,673	1,662,985	1.00
rRNA	468	168,909	0.06	346	50,383	0.03
tRNA	29	2,203	0.001	26	1,978	0.001
**Total**	282,447	29,842,011	11.09	90647	13103923	7.90
