# Supplementary material for: Reference-Guided De Novo Genome Assembly of the Flour Beetle Tribolium freemani
Source: Int J Mol Sci. 2022 May 24;23(11):5869. doi: 10.3390/ijms23115869 (PMC9180572; doi:10.3390/ijms23115869)
Supplement: Supplementary file 1 [file ijms-23-05869-s001.zip › Table S6.pdf]

Table S6. Abundance of DNA transposon subclasses in Tfree 1.0 and Tcas5.2 genome assemblies expressed as total number of specific element (graphical representation is given at the Figure 6C).

Class	Subclass	Tfree1.0	Tcas5.2	Difference
DNA transposon	Academ-1	940	573	367
DNA transposon	CMC-Chapaev-3	973	125	848
DNA transposon	CMC-EnSpm	41	12	29
DNA transposon	CMC-Transib	356	98	258
DNA transposon	Crypton	421	221	200
DNA transposon	Crypton-I	710	301	409
DNA transposon	Crypton-V	6	7	-1
DNA transposon	Ginger-1	3	3	0
DNA transposon	Ginger-2	11	4	7
DNA transposon	IS3EU	9086	3074	6012
DNA transposon	Kolobok-E	2469	901	1568
DNA transposon	Kolobok-Hydra	52	142	-90
DNA transposon	Kolobok-T2	16	3	13
DNA transposon	Kolobok-T2?	5	2	3
DNA transposon	MULE-MuDR	127	139	-12
DNA transposon	MULE-NOF	3286	1629	1657
DNA transposon	Maverick	1956	1537	419
DNA transposon	Merlin	11	7	4
DNA transposon	P	247	84	163
DNA transposon	PIF-Harbinger	54	15	39
DNA transposon	PIF-ISL2EU	36	2	34
DNA transposon	PIF-Spy	29	16	13
DNA transposon	PiggyBac	93	71	22
DNA transposon	Sola-1	70	8	62
DNA transposon	Sola-2	47	49	-2
DNA transposon	Sola-3	1	4	-3
DNA transposon	TcMar	47	40	7
DNA transposon	TcMar-Fot1	64	17	47
DNA transposon	TcMar-Mariner	602	325	277
DNA transposon	TcMar-Pogo	33	81	-48
DNA transposon	TcMar-Tc1	14387	10970	3417
DNA transposon	TcMar-Tc1?	574	608	-34
DNA transposon	TcMar-Tc4	61	20	41
DNA transposon	TcMar-Tigger	227	918	-691
DNA transposon	TcMar-m44	215	49	166
DNA transposon	TcMar?	3	2	1
DNA transposon	Zator	4	2	2
DNA transposon	hAT	7	3	4
DNA transposon	hAT-Ac	531	79	452
DNA transposon	hAT-Blackjack	97	71	26
DNA transposon	hAT-Charlie	123	55	68
DNA transposon	hAT-Pegasus	6	3	3
DNA transposon	hAT-Tag1	148	1	147
DNA transposon	hAT-Tip100	759	198	561
DNA transposon	hAT-hAT19	111	47	64
DNA transposon	hAT-hAT19?	1	1	0
DNA transposon	hAT-hATm	25	24	1
DNA transposon	hAT-hobo	33	18	15
