# Supplementary material for: Reference-Guided De Novo Genome Assembly of the Flour Beetle Tribolium freemani
Source: Int J Mol Sci. 2022 May 24;23(11):5869. doi: 10.3390/ijms23115869 (PMC9180572; doi:10.3390/ijms23115869)
Supplement: Supplementary file 1 [file ijms-23-05869-s001.zip › Table S7.pdf]

Table S7. Abundance and characteristics of organizational properties of the major satellite DNA TFREE in the *T. freemani* genome assembly Tfree1.0 and in the hifiasm contigs.

TFREE satellite DNA		
	**Tfree1.0**	**Hifiasm contigs**
Number of monomers	216,594	1,003,839
Total length (bp)	35,954,604	166,637,274
Abundance (%)	13.36	35.77
**Array (>800 bp or 5 consecutive monomers) statistics**		
Total number of arrays	2,766	
Min. length (bp)	812	
Q1 (bp)	5,833	
Median length (bp)	10,286	
Q3 (bp)	16,923	
Max. length (bp)	93,724	
Mean length (bp)	13,076	
