# Supplementary material for: Reference-Guided De Novo Genome Assembly of the Flour Beetle Tribolium freemani
Source: Int J Mol Sci. 2022 May 24;23(11):5869. doi: 10.3390/ijms23115869 (PMC9180572; doi:10.3390/ijms23115869)
Supplement: Supplementary file 1 [file ijms-23-05869-s001.zip › Table S8.pdf]

**Table S8.** Annotations of the *T. freemani* mitogenome and its comparison to the *T. castaneum* mitogenome (NC_003081.2). Alignment scores between *T. freemani* and *T. castaneum* mitochondrial genes were calculated using Needleman–Wunsch algorithm.

| Gene | Start position | End position | Size (bp) | Start codon | Termination | Similarity to *T. castaneum* mtDNA | |
|---|---|---|---|---|---|---|---|
| | | | | | | Alignment score | Similarity score |
| tRNA-Ile | 1 | 63 | 63 | | | 116,9696 | 0,984127 |
| tRNA-Gln | 61 | 129 | 69 | | | 97,33598 | 0,9275362 |
| tRNA-Met | 129 | 196 | 68 | | | 87,0156 | 0,9411765 |
| ND2 | 203 | 1207 | 1005 | ATA | TAA | 257,5144 | 0,7870647 |
| tRNA-Trp | 1206 | 1272 | 67 | | | 124,8967 | 0,9850746 |
| tRNA-Cys | 1272 | 1332 | 61 | | | 89,36294 | 0,9344262 |
| tRNA-Tyr | 1334 | 1397 | 64 | | | 1,497805 | 0,765625 |
| COX1 | 1394 | 2935 | 1542 | CAC | TAA | 1163,827 | 0,8463035 |
| tRNA-Leu | 2937 | 3002 | 66 | | | 74,40892 | 0,9090909 |
| COX2 | 3003 | 3687 | 685 | ATA | T | 544,5334 | 0,8510949 |
| tRNA-Lys | 3691 | 3761 | 71 | | | 132,8237 | 0,9859155 |
| tRNA-Asp | 3789 | 3855 | 67 | | | 29,10444 | 0,8358209 |
| ATP8 | 3856 | 4014 | 159 | ATC | TAG | -19,56359 | 0,754717 |
| ATP6 | 4008 | 4679 | 672 | ATG | TAA | 338,7267 | 0,8184524 |
| COX3 | 4679 | 5464 | 786 | ATG | TAA | 399,1438 | 0,8155216 |
| tRNA-Gly | 5467 | 5529 | 63 | | | 84,22575 | 0,9365079 |
| ND3 | 5590 | 5883 | 294 | ATC | TAG | -269,9518 | 0,5884354 |
| tRNA-Ala | 5882 | 5948 | 67 | | | 77,61037 | 0,8955224 |
| tRNA-Arg | 5948 | 6010 | 63 | | | 84,98782 | 0,952381 |
| tRNA-Asn | 6010 | 6074 | 65 | | | 79,08857 | 0,9384615 |
| tRNA-Ser | 6075 | 6133 | 59 | | | 69,63734 | 0,8983051 |
| tRNA-Glu | 6134 | 6198 | 65 | | | 64,5461 | 0,8923077 |
| tRNA-Phe | 6197 | 6261 | 65 | | | -184,3245 | 0,4461538 |
| ND5 | 6263 | 7976 | 1714 | ATA | T | 1040,293 | 0,827888 |
| tRNA-His | 7974 | 8039 | 66 | | | 19,24164 | 0,8030303 |
| ND4 | 8040 | 9372 | 1333 | ATG | T | 781,7487 | 0,8237059 |
| ND4L | 9366 | 9653 | 288 | ATG | TAA | 200,336 | 0,8368056 |
| tRNA-Thr | 9656 | 9719 | 64 | | | 86,2075 | 0,9375 |
| tRNA-Pro | 9720 | 9784 | 65 | | | 2,838774 | 0,8153846 |
| ND6 | 9790 | 10281 | 492 | ATT | TAA | 35,82355 | 0,7662602 |
| CYTB | 10281 | 11420 | 1140 | ATG | TAA | 595,9787 | 0,8192982 |
| tRNA-Ser2 | 11419 | 11485 | 67 | | | 61,39066 | 0,8955224 |
| ND1 | 11503 | 12456 | 954 | ATA | TAG | 700,5536 | 0,8427673 |
| tRNA-Leu2 | 12454 | 12518 | 65 | | | 64,54611 | 0,8923077 |
| l-rRNA | 12518 | 13762 | 1245 | | | 654,1954 | 0,8168675 |
| tRNA-Val | 13804 | 13872 | 69 | | | 113,0981 | 0,9565217 |
| s-rRNA | 13874 | 14648 | 775 | | | 702,1122 | 0,8696774 |
| Control region | 14649 | 15757 | 1109 | | | -763,1833 | 0,6723827 |
